# Effects of oncological care pathways in primary and secondary care on patient, professional and health systems outcomes: a systematic review and meta-analysis

**DOI:** 10.1186/s13643-020-01498-0

**Published:** 2020-10-25

**Authors:** Jolanda C. van Hoeve, Robin W. M. Vernooij, Michelle Fiander, Peter Nieboer, Sabine Siesling, Thomas Rotter

**Affiliations:** 1 Department Health Technology & Services Research, University of Twente, Enschede, the Netherlands; 2 Netherlands Comprehensive Cancer Organisation (IKNL), Utrecht, the Netherlands; 3 Department of Nephrology and Hypertension, University Medical Center Utrecht, Utrecht University, Utrecht, The Netherlands; 4 Julius Center for Health Sciences and Primary Care, University Medical Center Utrecht, Utrecht University, Utrecht, The Netherlands; 5 College of Pharmacy, Department of Pharmacology, University of Utah, Salt Lake City, USA; 6 Wilhelmina Hospital, Assen, the Netherlands; 7 School of Nursing, Queen’s University, Kingston, Canada

**Keywords:** Care pathways, Clinical pathways, Integrated care pathways, Care maps, Oncology, Cancer, Systematic review

## Abstract

**Background:**

Pathways are frequently used to improve care for cancer patients. However, there is little evidence about the effects of pathways used in oncological care. Therefore, we performed a systematic review and meta-analysis aiming to identify and synthesize existing literature on the effects of pathways in oncological care.

**Methods:**

All patients diagnosed with cancer in primary and secondary/tertiary care whose treatment can be characterized as the strategy “care pathways” are included in this review. A systematic search in seven databases was conducted to gather evidence. Studies were screened by two independent reviewers. Study outcomes regarding patients, professionals, and system level were extracted from each study.

**Results:**

Out of 13,847 search results, we selected 158 articles eligible for full text assessment. One hundred fifty studies were excluded and the remaining eight studies represented 4786 patients. Most studies were conducted in secondary/tertiary care. Length of stay (LOS) was the most common used indicator, and was reported in five studies. Meta-analysis based on subgroups showed an overall shorter LOS regarding gastric cancer (weighted mean difference (WMD)): − 2.75, CI: − 4.67 to − 0.83) and gynecological cancer (WMD: − 1.58, CI: − 2.10 to − 1.05). Costs were reported in six studies and most studies reported lower costs for pathway groups.

**Conclusions:**

Despite the differences between the included studies, we were able to present an evidence base for cancer care pathways performed in secondary/tertiary care regarding the positive effects of LOS in favor of cancer care pathways.

**Systematic review registration:**

PROSPERO CRD42017057592.

## Background

Care pathways are also known as “integrated care pathways,” “clinical pathways,” “critical pathways,” or “care maps” [1]. Care pathways are tools to guide evidence-based healthcare and have been implemented since the 1980s [2]. Care pathways provide a means to improve multidisciplinary communication and care planning, including primary and secondary/tertiary care. Further, pathways aim to improve communication between clinicians and patients as well as patient satisfaction [3]. In addition, care pathways are described to have a positive impact on quality of care, efficiency, and teamwork [4, 5]. Rotter et al. [6] conducted a systematic review on the effects of clinical pathways and concluded that clinical pathways are associated with reduced in-hospital complications and improved documentation without negatively impacting on length of stay and hospital costs. Although care pathways are frequently applied in cancer care, the evidence of its effects is often limited. Furthermore, most study designs which were used to evaluate pathways were relatively weak. To our knowledge, a systematic review of the effects of pathways in cancer care is not available [7].

Cancer care is complex and relies upon careful coordination between multiple healthcare organizations and providers. Technical information exchange and regular communication flow between all those involved in treatment (including patients, general practitioners, specialist physicians, and other specialty disciplines) is needed [8]. Therefore, care pathways are often used in cancer care and are seen as a method to provide patient-centered care, reduce waiting times, and improve quality of cancer care [9, 10].

The aim of this systematic review is to assess the effects of oncological pathways according to an unambiguous definition of cancer care pathway in studies providing a high level of evidence. In this systematic review, effects of cancer care pathways were assessed in comparison with usual care. In addition, an overview of the outcome measures regarding patients, professionals, and system level will be presented. Because cancer care is characterized by coordination and multidisciplinary communication within and between healthcare organizations, we searched for literature in primary as well as secondary/tertiary healthcare. Furthermore, information about the implementation of oncological care pathways was assessed. By conducting this systematic review and meta-analysis, we aimed to present the available high evidence in a substantiated and concise way, in order to improve the current evidence base regarding the effects of oncological care pathways.

## Methods

### Types of studies

We limited our study selection to the following study designs: randomized controlled trials (RCT), non-randomized studies (NRS), controlled before-after studies (CBA), and interrupted time series studies (ITS), as well as economic evaluations (cost-effectiveness analyses, cost-utility analyses and cost-benefit analyses, cost analysis, and comparative resource utilization studies), where available. Based on the suggested study designs of the Cochrane Effective Practice and Organisation of Care (EPOC) Group for inclusion in reviews, retrospective cohort studies, prospective cohort studies, cross-sectional studies, and case-control studies were excluded [11]. An additional file shows an overview of the inclusion criteria for this systematic review (see additional file 1).

### Types of participants

Eligible participants for inclusion in this systematic review were patients in primary and secondary/tertiary care which includes the coordination and continuity of healthcare as patients transfer between different locations or different levels of care. As potential patients, we considered all patients of every age and diagnosed with every type of cancer in primary and secondary care/tertiary care.

### Types of interventions

In this review, cancer care pathways were compared to usual care or care and treatment given to patients in a control setting. For the purpose of this review, we will define usual care as treatment determined at the discretion of the attending healthcare professional. This care may present the best current care, and may also be highly variable across different settings. Due to the different terminology used for cancer care pathway, we applied the definition of clinical pathways based on four operational pathway criteria: (1) multidisciplinary (two or more clinical professions involved), (2) protocol or algorithm based (i.e., structured plan/treatment-protocol or algorithm), (3) evidence based or based on practice guidelines, and (4) aiming to standardize cancer care [12]. Every pathway characteristic could be met as (1) “yes” criterion; (2) “not sure” because of poor reporting or when the authors did not reply to our emails and phone calls and therefore we were not able to retrieve more information about the study or (3) “criterion not met.” If one or more pathway criteria was not met, we excluded the study. In the “Results” section, additional information relating to the included studies and differences between the studies is presented.

### Types of outcome measures

Every measured patient, professional, and system level outcome was considered for inclusion. Patient outcomes include (in-patient) mortality, mortality at the end of follow-up, re-admissions (hospital setting), (in-hospital) complications, hospital admissions, adverse events, discharge destinations, performance status, patient satisfaction, quality of life, and absence from work. Professional outcomes include quality measures appropriate to the specific aim of the care pathway, staff satisfaction, team functioning, guideline adherence, and adherence to evidence-based practice. System level outcomes include length of stay, waiting times, costs, and hospital charges. Furthermore, any reported measure regarding implementation strategies and methods were also assessed.

### Information sources and search strategy

Systematic searches were performed in the Cochrane Library, Medline (1946-2019), Embase (1946-2019), Cinahl (1981-2019), Lilacs, ClinicalTrials.gov, and the World Health Organization International Clinical Trials Registry Platform, including conference abstracts. Because this systematic review aimed to present evidence regarding the effects of oncological care pathways, our literature search focused on “research” rather than “quality improvement.” Furthermore, grey literature was searched in Open Grey, the Grey Literature Report (1996–2017), and Open Clinical. Also organizational websites and professional organizations related to care pathways and implementation were assessed (European Pathway Association, National Health Service England, The National Institute for Health and Care Excellence (NICE), Cancer Council Australia, American Society of Clinical Oncology (ASCO), as well as the websites of: International Journal of Integrated care and Journal of Clinical Pathways).

Moreover, we employed citation tracking and examined included studies and previous reviews. We also contacted investigators to identify any study missed by the electronic searches. The most recent searches were conducted on July 1, 2020. Two reviewers independently screened all titles and abstracts (JvH, RV), using Covidence (www.covidence.org). A third reviewer (TR) was available for consultation in the case of disagreements between the two reviewers. The potentially relevant studies were further examined using full-text copies. All databases were searched from the date of inception forward with neither date nor language limits. See for the complete search strategy, additional file 2.

**Table:** 

Summary of the search strategy	
Pathway: Critical pathway, clinical pathway, patient care pathway, pharmacotherapeutic pathway, therapeutic pathway, treatment pathway, care plan, structured care, intensive management care, care algorithm, treatment algorithm, therapeutic algorithm, standardized (patient) care, standardized treatment, (care) map, process map	
Cancer: Oncology, neoplasm, carcinoma, malignant, tumor	
Oncology: Medical oncology, radiation oncology, surgical oncology, oncologist, radiation oncologist	
Guideline: Interdisciplinary guideline, cross disciplinary guideline, multidiscipline guideline, team guideline, standardized guideline, practice guideline	
Health professional: Clinician, provider, professional, doctor, nurse, family doctor, family physician, family practitioner, GP, practitioner, physician, hospital, pharmacy, primary care, regulatory, team	
Intervention: Intervention study, intervention care, intervention health, demonstration project, pre-test, post-test, improvement, impact, individualized, interdisciplinary, multicomponent, multidisciplinary, multifaceted, multimodal, personalized, standardized, usual care	
Study design: Randomized controlled trial, (controlled) clinical trial, placebo trial, quasi-experiment, experimental method, experimental study, experimental design, (interrupted) time series, multicentre study, controlled before-after study, interrupted time series analysis, evaluation study, prospective studies, retrospective study, meta-analysis, pilot project, systematic review, meta-nalysis, scoping review, concept analysis	

### Data collection process

From every included study, we extracted data regarding study characteristics, population characteristics, interventions characteristics, and outcomes.

Hospital costs and charges were assessed and calculated for the individual studies. Cost and charges data were calculated in US$ for the common price year 2016 by using the “CCEMG-EPPI-Centre Cost Converter” [13]. This Cost Converter is a web-based tool that can be used to adjust and estimate of cost expressed in one currency and price year to a target currency or price per year [13].

### Statistical analysis

For calculating the pooled effects estimate, called weighted mean difference (WMD), we used Review Manager from the Cochrane Collaboration [14]. To assess the comparability of the results from individual studies and included subgroups, we used the statistic for quantifying inconsistency: *I*^2^ = [(*Q* df)/*Q*] × 100%) [15]. We considered an overall test-value greater than 60% to serve as evidence of substantial heterogeneity of a magnitude where statistical pooling is not appropriate [15].

We used a random effects model since the model estimates the effect with consideration to the variance between studies, rather than ignoring heterogeneity by employing a fixed effects model.

Subgroup analysis based on differences in the setting, on the risk of bias of the studies, and on the age of the study population was not possible because these characteristics were not distinctive enough between the studies to form different subgroups.

### Quality assessment

Two reviewers independently assessed the quality of the studies (JvH, RV). Therefore, we adhered to the validated criteria suggested by the Effective Organisation of Care Group (EPOC) and defined three risk of bias classes: class I (low risk of bias), class II (moderate risk of bias), and class III (high risk of bias) [16]. A third reviewer was available for consultation in case of disagreements between the two reviewers (TR). To appraise the methodological quality of the included cost evaluations, the Evers checklist was used, which is recommended for Cochrane Reviews [17].

### Dealing with missing data

If a study did not provide information about the standard deviation, this was calculated based on the reported *p* value and mean difference. For calculating the standard deviation of the mean, we used the Revman Calculator (https://training.cochrane.org/resource/revman-calculator). By using this calculator, the assumption was made that the standard deviations of outcome measurements are the same in both study groups.

For calculating the mean as well as the standard deviation from the reported median and range, we used the mean variance calculator (http://www.comp.hkbu.edu.hk/~xwan/median2mean.html)

## Results

### Search results

The specialized search strategy led to 13,870 results. After removing duplicates, all of the 13,847 titles and abstracts were screened for inclusion. The remaining 158 possibly relevant studies were retrieved as full text articles. Based on the full text assessment, 150 studies were excluded. The majority of the excluded studies did not meet our study design criteria (79 studies). In addition, a number of studies compared different medical treatments and medication, the intervention did not meet our definition of cancer care pathways, or information was lacking (25 studies). Other excluded studies did not include cancer patients (15 studies), a control group was missing (15 studies), provided only an abstract or protocol (15 studies), full-text was not available (3 studies), or the outcomes described did not matched our inclusion criteria (1 study). Finally, eight studies matched our methodological requirements. In an additional file, the PRISMA flow diagram is presented (see Additional file 3). The excluded full text studies and the reason for exclusion are listed in Additional file 4. For the references of all excluded full text studies, see Additional File 5.

Below, we present the studies conducted in the secondary/tertiary healthcare setting separately from the study conducted in both primary and secondary healthcare, because the settings in which the studies were performed, intervention characteristics, and outcomes differed greatly.

### Quality assessment

Based on the validated criteria suggested by the Effective Organisation of Care Group (EPOC) [16], all studies were assessed as “high risk of bias,” except for two studies [18, 19]. In an additional file, the results of the risk of bias assessment are shown (see Additional file 6). The results of the cost evaluation according the Evers checklist [17] is presented in Additional file 7.

### Results of studies conducted in the secondary/tertiary healthcare (hospital care) setting

The majority of the included studies (seven studies) were conducted in the setting of secondary healthcare, within hospitals or in oncology centres [18–24]. These studies represented 1494 patients.

### Study characteristics

In this paragraph, a description in given of the following study characteristics: study designs, tumor location, sample size, country, and healthcare setting. In Table 1, the study characteristics of the included primary studies included are presented.

**Table 1:** Study characteristics of included primary studies

	Study ID	Study design	Tumor location	Sample Size	Country	Setting
1	Chen et al. 2000 [20]	Non-randomized controlled trial^a^ (with a historical control group)	Unilateral neck dissection	190	USA	Academic cancer Center, secondary/tertiary care
2	Dahl et al. 2017 [25]	Non-randomized controlled trial^a^ (with a historical control group)	Cancer patients (colorectal, lung, melanoma, breast, prostate, and other)	3292	Denmark	Danish hospitals, primary and secondary care
3	Gendron et al. 2002 [21]	Interrupted time series study^a^	Head and neck cancer surgery	212	USA	Tertiary care academic medical center
4	Ghosh et al. 2001 [22]	Interrupted time series study^a^	Hysterectomy cervical or endometrial cancer	151	USA	Academic Medical Center, tertiary care
5	Jeong et al. 2011 [23]	Non-randomized controlled trial^a^	Treatment of gastric cancer (early vs. advanced; non-CP vs. CP)	631	Korea	Unclear, secondary care
6	Kiyama et al. 2003 [18]	Randomized controlled trial	Gastric cancer	85	Japan	Nippon Medical School Hospital, secondary care
7	Tastan et al. 2012 [24]	Non-randomized controlled trial^a^	Breast cancer	69	Turkey	Military Medical Academy, secondary care
8	Williams et al. 2015 [19]	Randomized controlled trial	Pain screening and treatment in head and neck cancer	156	UK	Hospital, secondary care

*USA* United States of America, *CP* care pathway, *UK* United Kingdom

^a^Study design is not mentioned in the article; specification is based on the Cochrane study designs

#### Study designs

The specification of the study designs of the included studies were based on the description of the Cochrane Effective Practice and Organisation of Care (EPOC) Group [11].

We included two studies which applied randomized study designs [18, 19]. In these studies, patients were randomized to either a pathway group or a non-pathway group. Two studies used an interrupted time series study design (ITS); in these studies, a pre-pathway group was compared to two or more pathway groups [21, 22]. In one study, pathway groups at 12 and 36 months after implementation were compared to a pre-pathway group [21], and in the other study a pre-pathway group and pathway groups at 6, 12, and 18 months after implementation were used [22].

Furthermore, in three studies, a non-randomized controlled trial study design was applied [20, 23, 24]. In these studies, patients in the non-pathway group received general care, and simultaneously patient in the pathway group were managed based on the pathway. In one study, a historical control group was compared with two other groups; a pathway group and a non-pathway group [20].

#### Tumor location

In the articles, patients with different tumors were studied: three studies described the effects of pathways for head and neck cancer [19–21]. Other studies presented the results of pathways for gastric cancer [18, 23], gynecological cancer [22], and breast cancer [24].

#### Sample size

The number of included participants varied, and ranged from almost 70 patients to more than 600 patients [18–24].

#### Country

Three studies were conducted in Korea, Japan, and Turkey [18, 23, 24]. In addition, three studies were performed in the USA [20–22], and one in the UK [19].

#### Setting

Three studies were conducted in general and non-academic hospitals or oncology centres [18, 19, 24]. Other studies were performed in an academic hospital [20–22]. In one study, the setting was not clearly reported [23].

#### Population characteristics

The population characteristics of the study groups included in the studies was listed in Table 2. All studies, expect one [22], reported patient characteristics on gender and age. Two studies reported also characteristics about socioeconomic status [21, 24].

**Table 2:** Population characteristics of included primary studies

	Study ID	Study groups	Gender (male vs female)	Age	Socioeconomic status
1	Chen et al. 2000 [20]	Historical control group nonpathway group Pathway group	76% vs.. 24% 64% vs. 36% 73% vs. 27%	58 years (median) 59 years (median) 60 years (median)	No information available
2	Dahl et al. 2017 [25]	Before implementation After implementation total After implementation pathway referred After implementation non-pathway referred	45% vs. 55% 52% vs. 48% 49% vs. 51% 54% vs. 46%	11.3/14.8/25.3/28.5/20.2^a^ 7.0/12.2/24.9/33.1/22.8^a^ 6.4/10.4/25.5/33.2/24.6^a^ 7.3/13.3/24.6/33.1/21.6^a^	No information available
3	Gendron et al. 2002 [21]	Control group (pre-pathway) 1 year after pathway implementation 3 years after pathway implementation	71% vs. 29% 79% vs. 21% 73% vs. 27%	65 years (median) 61 years (median) 60 years (median)	Smoking (yes): 96%; alcohol use (yes): 75% Smoking (yes): 90%; alcohol use (yes): 73% Smoking (yes): 90%; alcohol use (yes): 54%
4	Ghosh et al. 2001 [22]	Separate groups for cervical and endometrial cancer Preintervention group Postintervention group Postintervention group Postintervention group	No patients characteristics were reported. Patients were matched for age, comorbid conditions, and stage of disease only.		
5	Jeong et al. 2011 [23]	Non care pathway group early gastric cancer Pathway group early gastric cancer Non care pathway group advanced gastric cancer Pathway group advanced gastric cancer	71% vs. 29% 64% vs. 36% 69% vs. 31% 65% vs. 35%	59.7 (mean) 58.2 (mean) 59.1 (mean) 59.3 (mean	No information available
6	Kiyama et al. 2003 [18]	Traditional care group Clinical pathway group	66% vs. 34% 62% vs. 38%	66.8 years (mean; ± 12.9) 63 years (mean; ± 12.1)	No information available
7	Tastan et al. 2012 [24]	Control group Clinical pathway group	No information available	53.2 (mean; ± 12.3) 51.7 (mean; ± 11.3)	Marital status (married vs. single): 82.4% vs. 17.6; ownership child (no vs. yes): 11.8% vs. 88.2; education (primary/secondary/high school/college or higher): 8.8%/50%/17.6%/23.6%; occupation (yes vs. no): 32.4% vs. 67.6%. Marital status (married vs. single): 82.9% vs. 17.1; ownership child (no vs. yes): 2.9% vs. 97.1; education (primary/secondary/high school/college or higher): 11.4%/45.7%/28.6%/14.3%; occupation (yes vs. no): 17.1% vs. 82.9%.
8	Williams et al. 2015 [19]	Usual care group Intervention group	64% vs. 36% 66% vs. 34%	58 years (mean; range 19-80) 60 years (mean; range 39-82)	No information available

^a^The information about age was reported in the following categories: 18–44 years; 45–54 years; 55–64 years; 65–74 years; ≥ 75 years

### Intervention characteristics

In this paragraph, the intervention characteristics reported in the studies were described: study groups, intervention, and care pathway. See Table 3 for more detailed information.

**Table 3:** Intervention characteristics of included primary studies

	Study ID	Study groups	Intervention	Care pathway^a^	Outcomes
1	Chen et al. 2000 [20]	Historical control group (prepathway, Sep 1993–Dec 1994) Contemporaneous nonpathway group (Sep 1996-Aug 1998) Clinical pathway group (Sep 1996-Aug 1998) Patients underwent the same surgical procedure during the time of implementation, but were not managed based on the pathway. The treated physician decided solely to place patients on the pathway.	The neck dissection pathway was presented in a tabular format and consists of the following aspects: assessment/evaluation, consult, diagnostic test, treatment, medication, performance status/activity, nutrition, teaching/psychosocial, discharge planning, outcome criteria and follow-up criteria. The activities were described for the initial evaluation, preoperative visit, and same day admit surgery.	Meets criteria 1–4	Length of hospital stay (median) Complications Readmission Costs of care
2	Dahl et al. 2017 [25]	Before implementation (Sep 2004–Aug 2005) After implementation total (May–Aug 2010) After implementation pathway referred (May–Aug 2010) After implementation non-pathway referred (May–Aug 2010)	The framework of the Danish cancer pathways includes three different descriptions of the pathway: a flowchart, a narrative text and a table providing an organizational overview. A pathway in the Danish context is a standardized pathway that most patients suspected of cancer will be able to follow. It describes the patient’s pathway from clinical suspicion of a certain cancer through diagnostic procedures and treatment. The pathway describes the medical procedures, the necessary organization encompassing both primary and secondary sectors of the health system, and timeframes in accordance with the political agreement. Main emphasis in the pathways are on information to be given to the patient, explicit identification of the responsible health professional or department in all phases, procedures for referral, description of multidisciplinary teams in each pathway as a forum for decisions on diagnosis and recommended treatment, and timeframes of all phases. An example of a pathway is shown [Probst et al. 2012].	Meets criteria 1–4	Patient dissatisfaction with long term waiting times
3	Gendron et al. 2002 [21]	Control group (pre-pathway) (1995) 1 year after pathway implementation (July 1996–July 1997) 3 years after pathway implementation (1999)	The pathway for patients undergoing major resection for upper aerodigestive tract cancer was implemented in July 1996. The format for the pathway is a 1-page table containing a list of goals and interventions for each postoperative day, followed by a page for each day on which accomplishments are recorded. When goals were not met, the variances are recorded in detail on the flow sheet.	Meets criteria 1–4	Length of stay (median, range) Readmission Complication rates Hospital charges
4	Ghosh et al. 2001 [22]	Separate groups for cervical and endometrial cancer: Preintervention group (Jan 1997–June 1998) Postintervention group (July 1998–Dec 1998) Postintervention group (Jan 1999–June 1999) Postintervention group (July 1999–Dec 1999)	Care pathways for patients with gynecologic malignancies were developed based on the results of clinical trials and on the consensus of experts. The pain control team and a pharmacist were involved. The nursing team played an active role in the practicality of the execution of these care plans. Documentation including preprinted orders were created and approved by hospital committees. Postoperatively, patients were placed on preprinted orders, which addressed patient education, rapid diet advancement, a reduction in laboratory tests, deep vein thrombosis prophylaxis, and pain management.	Meets criteria 1–4	Length of hospital stay (mean, SD) Total costs Direct costs Patient satisfaction Readmission rates
5	Jeong et al. 2011 [23]	Separate groups for early gastric cancer and advanced gastric cancer: Non care pathway (general care) group Pathway group Both groups: Dec 2006-Nov 2007	The pathway was first implemented in September 2004. The pathway for patients with gastric cancer following gastrectomy were developed in 2006 to provide care for these patients. The pathway was electric medical record based. In the pathway for hospital staff which is presented in figure 1, the aspects: Lab, Treat, Activity, Diet, Mx, Education, and Evaluation were described for the day before surgery, the day of surgery until 2/3 days after surgery (preoperative laboratory tests and diagnostic modalities, assessment of concomitant diseases, consultation for operative safety, bowel preparation and antibiotics at preoperative day 1 and postoperative day 1, removal of nasogastric tube, start of semi-fluid diet, removal of closed suction drain before discharge. There is also a pathway for patients; this is presented in Fig. 2.	Meets criteria 1–4	Length of hospital stay (pre, post and total) (mean) Costs (pre, post and total)
**6**	Kiyama et al. 2003 [18]	Traditional care group (control) Clinical pathway group Both groups: January 2001 to December 2001.	The CP employed standardised postoperative management using printed order sets, which included instructions for such matters as medication, diet, removal of the catheter and the mobility of the patients.	Meets criteria 1–4	Length of hospital stay: pre- and postoperative (mean, SD) Morbidity rate Postoperative complications
**7**	Tastan et al. 2012 [24]	Control group (clinical pathway was not used) Clinical pathway group Both groups: March 2004-April 2005	The clinical pathway was constructed after conducting a literature survey. The clinical pathways were organized to make them suitable for the clinic by considering work order and resources of the clinic along with the doctors and nurses. For this study, a standard clinical pathway that included possible problems of the patient, clinical goals, and the medical team’s interventions for reaching the treatment goals was designed. Primary components of the breast surgery care protocol are: consultation/visit (physician, anesthetist, and nurse), diagnostic processes, patient evaluation/diagnosis processes, medication, treatment and clinical procedures, diet, activity/security, and psychological/educational/discharge planning (Appendix 1). This was described for the admission day, the operation day and the postoperative days 1 until 4.	Meets criteria 1 - 4	Patient anxiety Quality of life Patient satisfaction
**8**	Williams et al. 2015 [19]	Usual care group Intervention group Both groups: Feb 2011-Jan 2013 The usual care treatment is based on the Royal Marsden Hospital Pain and Palliative Care treatment guidelines. The intervention group received combined screening, treatment and educational approach. Patients in the usual care group were not proactively assessed at baseline, nor did they receive a timetabled weekly pain assessment conducted by their pain physician They also did not receive the pain education brochure.	Pain assessment and treatment was conducted by two pain clinic doctors and two nurses who were independent of the research team. Treatment took place immediately after allocation to the intervention group, and continued throughout the three month study period. Treatment was individualized according to analgesic needs and requirements according to the Royal Marsden Hospital Palliative Care & Pain Control guidelines, which are based on the WHO and British Pain Society guidelines. First the initial consultation took place. Further, follow-up sessions took place weekly either by telephone or in a pain clinic consultation. Each patient was also given an educational brochure about cancer pain and its treatment and this was discussed with a control pain doctor at the baseline time point. Subjects were asked proactively about their suitability for these additional pain control treatments. Different analgesic drugs and their expected benefit and side-effects were discussed.	Meets criteria 1–4	

*CP* care pathway, *UK* United Kingdom, *SD* standard deviation

^a^The described pathway was defined using the working definition of “care pathways”:

1. The intervention was a structured multidisciplinary plan of care

2. The intervention was used to translate guidelines or evidence into local structures

3. The intervention detailed steps in a course of treatment or care in a plan, pathway, algorithm, guideline, protocol or other “inventory of actions” (i.e., the intervention had time-frames or criteria-based progression)

4. The intervention aimed to standardize care for a population of cancer patients

An intervention is considered to be a care pathway if it meets all four criteria

The specific interventions regarding the pathways described in the included studies showed considerable variation. Most studies focused on pathways for the perioperative phase in order to guide surgical management [18, 20–24], and one study investigated pain management [19]. In the studies focusing on for surgical care, the following key components which were addressed in these pathways were described: nutrition and diet [18, 20, 22–24], diagnostic modalities, and laboratory tests [20–24]; medication [18, 20, 23, 24]; patient education [18, 22, 24]; preoperative consultation and visits [20, 23, 24]; drains [21, 23]; activity [20, 24]; clinical procedures and treatment [20, 24]; discharge planning or instruction [20, 24]; assessment and preadmission testing and evaluation [20, 23]; and psychosocial support and education [20, 24]. Other components which were mentioned in one study only: performance status, outcome criteria, follow-up criteria, and follow up care [20]; pain management and pain control; and deep vein thrombosis prophylaxis [22], preoperative bowel preparation and fasting, and removal of nasogastric tube [23], removal of a catheter and mobility [18].

The study included in this review investigating pain management, included an initial consultation with a control pain doctor and weekly follow up sessions [19].

In addition, in three studies the pathway was presented in detail providing a description of the pathway as well as a figure of the pathway [20, 23, 24]. Further, one study presented a pathway for the hospital staff as well as a pathway for patients [23]. In the other studies, no detailed information about the pathway was available.

#### Outcomes

The most frequently used patient outcomes reported in the included studies were complications [18, 20, 21] and readmission [20–22]. Other reported patient outcomes were patient satisfaction [19, 24], patient anxiety [19, 24], morbidity [18], and quality of life [24]. However, these quality outcomes measures were not comparable between the studies.

Professional outcomes such as staff satisfaction and team functioning were not reported in the included studies. Furthermore, LOS was the most common used indicator for system level outcomes and was reported in five studies [18, 20–23]. For this outcome measure, we were able to carry out a meta-analysis; see the paragraph about Effects on Length of Stay. Other system level indicators which were reported focused on costs and hospital charges [18–23], but these studies showed considerable differences in definitions and results. Nevertheless, in most studies, the actual costs instead of charges were reported [18–20, 22, 23], because costs are set constant over time. In one study the median total charges per patient was used as the primary outcome [21]. In addition, in all studies in which costs were reported, fixed as well as variable costs were included in the total costs. Besides, the studies showed differences in aspects which were included in the total costs. In four studies, patient visits, consultation, assessment, and diagnostic- and laboratory tests, as well as treatment were included [18–20, 22]. Medication was included in five studies [18–22]. Facilities, like inpatient ward costs, operation room, medical and surgical supplies were reported in two studies [21, 22]. One study included professional fees [20]. And in another study, the costs for patient monitoring and patient education were included [22].

Additionally, two studies reported extra information about the methods for conducting the cost analysis: in one study, the hospital and professional costs were combined into a model that has been developed to set costs constant over time [20] and in another study, quality adjusted life days (QALD’s) were generated and the results were presented in a Cost Effectiveness Acceptability Curve (CEAC) related to the willingness to pay [19].

See Table 3 for a summary of the outcomes reported in the studies.

### Effects on patient outcomes

**Fig. 1:**
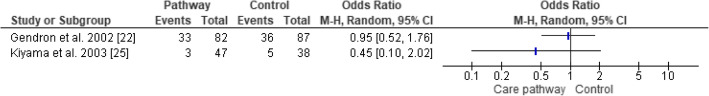
Reported complications

**Fig. 2:**
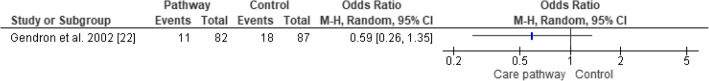
Reported readmissions

Patient outcome measures were reported in four studies [18, 20–22]. However, only two studies reported the measured effects in terms of complications [18, 21], and one study reported outcomes measures in terms of readmissions [21]. Therefore, statistical pooling of quality outcomes could not be performed. Both studies reporting effects of complications described less observed complications among the pathway groups [18, 21]. The study reporting effects of readmissions described less readmissions for the pathway group within 30 days after surgery [21]. See for the results of the reported complications in Fig. 1 and the reported readmissions in Fig. 2.

### Effects on length of stay

**Fig. 3:**
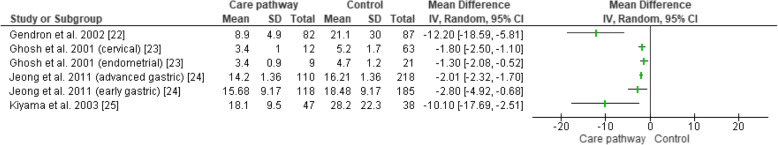
Reported effects on LOS

The effects of cancer care pathways on LOS were reported in five studies [18, 20–23]. All included studies that measured LOS reported results in favor of cancer pathways. In two studies, both mean and standard deviation were reported [18, 22], and three studies did not provide information on the standard deviation [20, 21, 23]. One study reported the mean LOS, and the SD was calculated by using the Revman Calculator (https://training.cochrane.org/resource/revman-calculator). In another study, the median and range was reported and the mean as well as the standard deviation were estimated [21] (see “dealing with missing data” in the “Methods” section). In one study, the median was reported only [20]; therefore, we were not able to calculate the mean and standard deviation. Two studies consisted of two subgroups which were separately studied [22, 23]. See Fig. 3 for the results of all reported effects on LOS.

After conducting a meta-analysis with data of four studies [18, 21–23], which represented a study population of 1079 patients, substantial heterogeneity between the studies was observed (*I*^2^ = 72%). Therefore, a forest plot with the total results without the pooled effects of all included studies reporting on LOS was presented in Fig. 3. The results of the meta-analysis of subgroups are presented in the section “Subgroup analysis.”

### Effects on costs

Out of nine studies, six studies reported on costs effects [18–23]. In four studies, including two studies with each two subgroups, lower costs were reported for pathway groups [18, 20–22], and three of these studies reported a significant reduction of costs related to cancer care pathways [18, 20, 23]. However, in one subgroup of a study, the total hospitals costs and the preoperative costs were lower in the pathway group, but the postoperative costs were higher in the pathway group [23]. Another study reported lower total and medication costs, but higher total daily costs in the pathway group [18].

Nevertheless, we observed a considerable methodological variation regarding the different methods used for cost calculation. In some studies, a full cost approach was used, whereas other studies included only direct hospital costs. In Table 4, the costs differences are presented. In addition, we have provided the un-discounted cost data in a separate table shown in Additional file 8, to allow readers recalculate the results using any discount rate.

**Table 4:** Cost/charges data, standardized to the year 2016 (CCEMG EPPI tool used)

Study ID	Country	Currency	Costs included	Pathway	Control	Reduction of costs, per patient
Chen et al. 2000 [20]	USA	US$	Total costs including hospital and professional fees: surgery-related costs, treatment-related costs, medications, consultations, and assessment and diagnostic tests.	$8448.62	$11,476.93 (historical control group, HCG) $9341.37 (non-pathway group, NPG)	HCG vs. pathway: − $3028.31 NPG vs. pathway: − $892.75
Gendron et al. 2002 [21]	USA	US$	The charge summary was divided into the following 6 categories: total, hospital room, pharmacy, operating room, laboratory, and other charges. Professional fees were not included.	$103,160.57 (> 1 year, group 1) $86,155.35 (> 3 years, group 2)	$137,769.62	Control vs. pathway group 1: − $34,609.05 Control vs. pathway group 2: − $51,614.27
Ghosh et al. 2001 [21] (cervical cancer)	USA	US$	Direct costs were obtained including hospitalization, pharmacy, laboratory, operation room, radiological, and other miscellaneous costs (the last includes: physical therapy, respiratory therapy, patient monitoring, and patient education).	$5204.43	$7361.88	− $2157.45 (− 29%)
Ghosh et al. 2001 [21] (endometrial cancer)	USA	US$		$5031.83	$6327.63	− $1295.80 (− 32%)
Jeong et al. 2011 [23] (advanced gastric cancer)	Korea	US$	Total hospital costs There is no description available of which costs are included.	$9297.65	$9329.28	− $31,63
			Preoperative costs	$1330.75	$1651.92	− $321.17
			Postoperative costs	$7966.90	$7681.14	+ $285.76
Jeong et al. 2011 [23] (early gastric cancer)	Korea	US$	Total hospital costs There is no description available of which costs are included.	$9997.61	$11,119.04	− $1121.43
			Preoperative costs	$1475.00	$1975.97	− $500.97
			Postoperative costs	$8522.61	$9143.07	− $620.46
Kiyama et al. 2003 [18]	Japan	US$	The total costs The total direct costs reported were the total medical costs (including medication and examinations).	$13,380.86	$17,206.63	− $3825.77
			Medication costs only	$1695.01	$2410.03	− $715,02
			Daily total costs	$519.91	$495.58	+ $24.33
Williams et al. 2015 [19]	UK	US$	Costs included: analgesic drug costs, pain clinic visits, use of physiotherapy, psychology and other resources.	$629.64	$336.79	+ $292.85

*USA* United States of America, *US*$ United States Dollar, *UK* United Kingdom

### Implementation of cancer care pathways

Information about the implementation of pathways was reported in five studies [20–24]. To categorize the detailed information about the reported implementation process of the pathways, we used the refined taxonomy for guideline implementation of Mazza and colleagues [26]. This taxonomy was based on the Cochrane Effective Practice and Organisation of Care (EPOC) data collection checklist and developed to classify the nature and content of implementation strategies. The taxonomy consisted of four domains: professional, financial (healthcare professionals, patients), organizational (healthcare professionals, patients, structural), and regulatory.

#### Professional domain

##### Present materials at meetings

In two studies, healthcare professionals were given information regarding the pathway in order to implement the pathway adequately [20, 24]. Also, several conferences and seminars were organized for outpatient and inpatient healthcare teams working with a disease site working group. Further, physicians were briefed on the pathway [20]. In another study, nurses underwent a 2-h training session to refresh their information on cancer risk factors, symptoms, diagnostic methods, treatment, pre-operative and post-operative nursing care for patients, and discharge procedures. In addition, doctors and nurses were given information about the clinical pathway and their duties and responsibilities while implementing it [24].

#### Organizational domain

##### Creation of an implementation team

In three studies, a multidisciplinary group was involved in the development of the pathway [20, 21, 23]. In one study, a core group determined which pathways were developed and a disease site working group was organized to draft the pathway [20]. In another study, the pathway was developed and continued to be modified by a multidisciplinary team which included surgeons, nurses, and allied healthcare representatives [21]. In addition, in one other study, the involvement of the multidisciplinary team was less clear [22]. In this study, the development of the pathways within a multidisciplinary team was not mentioned, but the pathways were based on the results of clinical trials and consensus of experts. Furthermore, these pathways were developed in cooperation with the department of anesthesia pain service and a pharmacist reviewed the recommendations. Moreover, the nursing team played an active role in developing these pathways [22].

##### Change in information and communication technology

In one study, it was described that almost 1 year after the pathway was implemented, an electronic medical record (EMR)-based care pathway was being used [23].

No implementation activities in the regulatory and financial domains were described in the primary studies.

### Subgroup analyses

Subgroup analyses were conducted in order to formulate more thorough conclusions relevant for clinical practice. These analyses were performed according to the protocol described previously.

#### Type of tumor

**Fig. 4:**
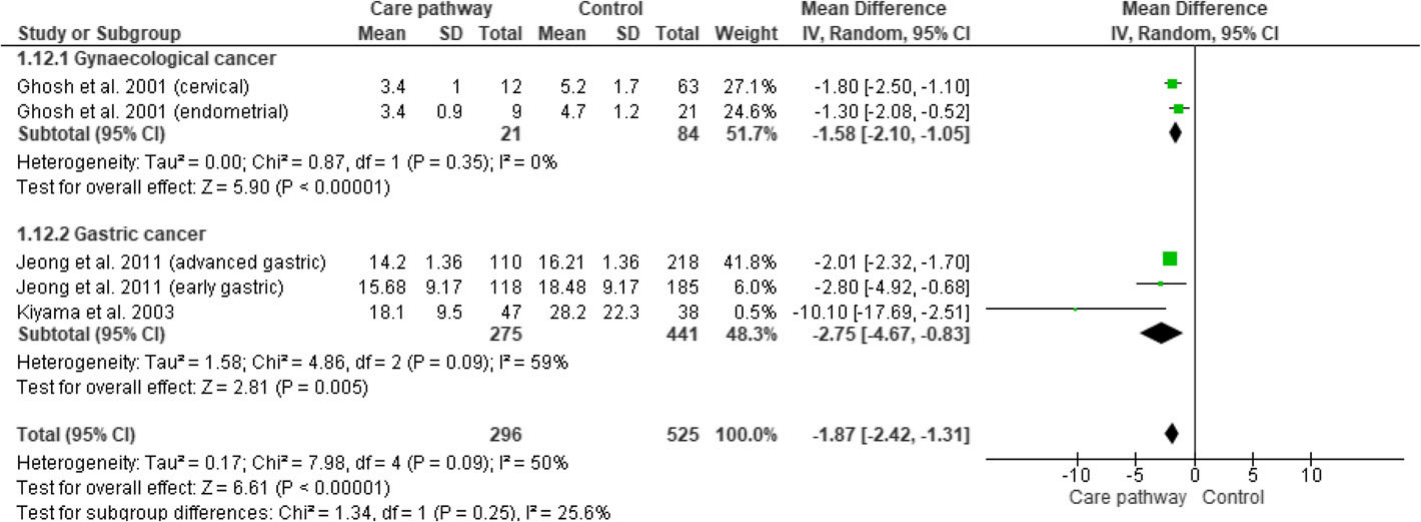
Subgroup analyses of the effects on LOS

The included studies were subdivided by type of tumor. We created subgroups of the study with subgroups including patients with gynecological cancer [22] and studies including patients with gastric cancer [18, 23]. Based on the random effects model, pathways for patients with gastric cancer showed a statistical significant pooled reduction of more than two and a half days compared to usual care (WMD: − 2.75; CI: − 4.67 to − 0.83). In the study with subgroups including patients with cervical and endometrial cancer, we observed a statistical significant pooled LOS reduction of more than one and a half day (WMD: − 1.58; CI: − 2.10 to − 1.05). Furthermore, the total pooled LOS reduced almost 2 days (WMD: − 1.87; CI: − 2.42 to − 1.31), which was a statistical significant result, associated with a moderate amount of heterogeneity (*I*^2^=50%). Nevertheless, it should be mentioned that the effects on LOS for pathways regarding patients with gynecological cancer was based on one overall study, which contained two subgroups. See Fig. 4 with the subgroup analyses of the effects on LOS.

#### Country

The primary studies were ordered by country to examine possible different market effects. Therefore, studies carried out in North America versus the studies performed in Asia (Korea and Japan) were analyzed. However, no association between country and the impact of pathways on LOS was detected.

#### Year of publication

Subgroup analysis based on the year of publication was similar for the subgroups subdivided by country, which showed no association.

### Sensitivity analysis

First, the robustness of the pooled LOS effects was determined by the available information about the standard deviation on LOS. In two studies, both mean and standard deviation were reported [18, 22], and for three studies the standard deviation was calculated due to missing information [20, 21, 23], see “Effects on length of stay” section.

We tested the robustness of the pooled LOS effects using different statistical calculation models, i.e., fixed versus random effects model. The pooled effects changed slightly when using the fixed effects model which indicates reliable pooled results.

In addition, sensitivity analysis was performed to test whether the effects size of LOS varied by the countries were the studies were carried out. Subsequently, we tested the hypothesis that different market forces are possibly confounding the conclusions of our meta-analysis. After stepwise exclusion of the studies carried out in North America, the pooled LOS effect increased, while the statistical heterogeneity reduced (WMD: − 2.75; *I*^2^ = 59%).

Sensitivity analysis were also performed to analyze the variation in the year of publication in order to test our hypothesis that pathways which were developed and implemented in more recent years could have had more success in reducing the LOS than less recent studies (or vice versa). After stepwise exclusion of all studies published before 2003, one study with two subgroups showed no statistical heterogeneity and a statistical significant pooled LOS effect of − 2.03 days.

### Results of studies conducted in the primary and secondary healthcare (hospital care) setting

#### Study characteristics

This paragraph provides a description of the following study characteristics: study designs, tumor location, sample size, country, and healthcare setting of the study covers both the primary setting as well as the secondary setting [25]. This study used a non-randomized controlled trial study design. A control group was compared to two post-pathway groups, i.e., a pathway group and a non-pathway group. The study focused on cancer pathways for patients with colorectal cancer, lung cancer, breast cancer, prostate cancer, melanoma, and other types of tumors. This study was performed in Denmark and represented 3292 patients [25] (see Table 1).

#### Population characteristics

Information about the group of patients before the implementation of pathways and three groups after implementation of the pathways was available regarding gender and age. No information was available about the socioeconomic status. See Table 2 for more information.

#### Intervention characteristics

This study was based on the implementation of standardized cancer patients’ pathways in 2008. Therefore, dissatisfaction of cancer patients with long waiting times was investigated. Although the cancer pathways did not include the diagnostic workup performed in general practice, the study focused on the time between referral by patients’ general practitioner (GP) to the first consultation at the hospital. Besides using registered data, patients were questioned about their satisfaction with the waiting times and GP’s were questioned about their involvement in diagnosing the cancer. In the Danish healthcare system, the GP serves as a gatekeeper to secondary care and GP’s refer patients to other clinicians when there is a reasonable suspicion of cancer. The authors concluded that the waiting time during the diagnostic process was reduced and patients were more satisfied after implementation of cancer pathways [25].

More detailed information about the specific pathway which was studied was found an article included in the list of references [27]. In this publication, it was stated that a pathway in the Danish context is a standardized pathway that most patients suspected of cancer will be able to follow. It describes the patient’s pathway from clinical suspicion of a certain cancer through diagnostic procedures and treatment. The pathway describes the medical procedures, the necessary organization encompassing both primary and secondary sectors of the health system, and timeframes in accordance with the political agreement. Main emphasis in pathways is placed on information to be given to the patient, explicit identification of the responsible healthcare professional or department in all phases, procedures for referral, description of multidisciplinary teams in each pathway as a forum for making decisions on diagnosis and recommended treatment, and timeframes of all phases [27]. In addition, the framework of the Danish cancer pathways includes three different descriptions of the pathway: a flowchart, a narrative text and a table providing an organizational overview. An example of a Danish cancer pathway was presented [27]. See Table 3 for more information about the intervention characteristics.

#### Outcomes

Patient satisfaction was the reported measured, which was measured using a patient questionnaire and a general practitioner questionnaire. This information was supplemented with register data [25].

### Quality assessment

To assess the quality of this study conducted in both primary and secondary, we adhered to the validated criteria suggested by the Effective Organisation of Care Group (EPOC); see “Results studies in the secondary/tertiary care setting, quality assessment” in the “Result” section [16]. This study was assessed as being at high risk of bias (Additional file 6).

### Implementation of cancer care pathways

The included study described the development of cancer care pathways rather specific, but little information was given about the implementation process. Based on the description regarding the development, we may assume that there was multidisciplinary involvement of clinicians and other healthcare professionals. But it remains unclear which role the regional representatives and other relevant healthcare professionals had in the implementation process of the pathway.

Using the taxonomy for guideline implementation [26], the organizational domain was addressed for at least the development of the presented pathways.

#### Organizational domain

##### Creation of an multidisciplinary team

In this study, the development of cancer care pathways was based on a common framework. Subsequently, healthcare professionals formulated the medical content in accordance with the consensus-based framework and finally the pathways were approved by a two-step process involving all stakeholders. This framework ensured that all stakeholders were able to influence the process which could be characterized as a “bottom-up and top-down” approach with involvement of both local and central actors, and in which administrators, healthcare professionals, and politicians cooperated to strike a balance. Further, agreeing on a framework and the integration of needs from the view of various professional disciplines, created a common understanding on how the best possible pathway was acquired. The framework was used for all cancer types to ensure consistency and ease the implementation of the various pathways.

In addition, for the development of these pathways, working groups developed the content using a consensus-making process where all stakeholders participated actively and contributed to the final product. The clinical working groups were asked to describe standard timeframes for the various elements involved in each pathway. These timeframes were further estimated without consideration of existing capacity and resources and thus indicate the minimum time needed to treat an “ideal patient” in an “ideal health system.”

Once a pathway had formally been approved, the five health regions had three months to ensure implementation at the local level. The regional representatives in the working groups knew the pathways in details which was an important factor in ensuring the implementation process [27].

## Discussion

We screened more than 13,000 published studies to assess the effects of cancer care pathways in primary and secondary/tertiary care. Finally, eight studies met our inclusion criteria with a total of 4786 patients. The included studies were conducted in six different countries and the investigated care pathways covered for more than 10 different types of tumors in general hospitals or academic hospitals in the primary and secondary healthcare setting.

Most studies were conducted in secondary/tertiary care and concerned the perioperative surgical care process. One study was carried out in both primary and secondary care, measuring dissatisfaction with waiting times. However, we observed considerable variance between the included studies regarding the pathways which were measured, the settings in which the studies were performed as well as the reported outcomes, especially the reported costs. Despite these differences, all included studies that measured LOS reported results in favor of cancer care pathways. Further, as a consequence of the clinical variability between the included studies, we observed a considerable statistical heterogeneity and therefore meta-analysis was often inappropriate. However, we were able to calculate the pooled effects of LOS for subgroups based on type of tumor and observed positive impact of cancer care pathways for patients with gastric and gynecology cancer which can be of interest for clinicians and managers.

In order to answer the secondary research question, we collected and analyzed information about the implementation of cancer care pathways in the included studies. In more than half of the included studies, implementation activities regarding cancer care pathway were described. Almost all these activities could be categorized as “professional” aspects (such as presenting materials in order to inform healthcare professionals, educating groups, and providing feedback) and “organizational” aspects (such as creating a multidisciplinary team, and changing the information and communication system). We observed that all studies which reported a positive impact on LOS in favor of the pathway described the involvement of a multidisciplinary team in the development of the pathway. Based on these observations, it is likely that the involvement of a multidisciplinary team could be a success factor in achieving positive outcomes of cancer care pathways. In addition, literature confirmed that implementation strategies have been poorly reported and evidence on successful clinical pathway implementation is limited and varies significantly in how healthcare organizations implement pathways [28]. Due to the differences in activities which were used for implementing care pathways, we could not formulate thorough conclusions about implementation strategies related to the differences in outcomes based on this review.

Although we searched for studies in primary and secondary/tertiary healthcare, we mainly found studies performed within secondary cancer care and only one study was related to both primary care and secondary healthcare. A possible reason why we did not find many publications in the primary care setting could be because pathways are widely used in inpatient and secondary/tertiary care, but their potential benefit in primary care is largely unclear [12].

This systematic review has several limitations. Despite our electronic search strategy, the additional search in grey literature and the independent screening of the search results, it is possible that some studies are overlooked. In addition, in order to present an evidence base regarding the effects of oncological care pathways, we focused in our literature search on “research” rather than “quality improvement,” which may have contributed to publication bias. However, we included all studies meeting our definition of care pathways, also when the term pathway was not mentioned in the text. Furthermore, due to the clinical variability of the included studies as well as the low number of included studies reporting on the outcome measures, the pooled effects on LOS should be interpret carefully.

Furthermore, from the perspective of clinical relevance, the correlation between the presence of complications and readmission is interesting, because patients can be readmitted to the hospital after a complication occurs. In addition, there is evidence that complications are a risk factor for in hospital readmissions [29]. However, we did not find any results of correlation analysis on this in the included studies.

## Conclusion

This systematic review was conducted in order to identify, assess, and synthesize all quantitative studies on the effects of oncological care pathways. Despite of the differences between the included studies, we were able to present an evidence base for cancer care pathways regarding the subgroup effects of LOS. However, the effects on complications, readmissions, and costs as well as the implications of differences in implementation of cancer care pathways were not conclusive enough.

Finally, cancer care pathways have shown their value for clinical practice; however, a comparison of care pathways is challenging and the impact of the implementation process on the outcomes remains rather unclear. Because of the paucity of high-quality evidence on the important questions covered by the review, there is a need for further well-designed research and audit.

## Supplementary information

**Additional file 1:.** Overview of inclusion criteria for this systematic review.

**Additional file 2.** Care pathways search strategy Ovid Medline: 1946 to July 1, 2020.

**Additional file 3.** Systematic review cancer care pathways PRISMA flow diagram.

**Additional file 4.** Excluded full text studies with the reason for exclusion.

**Additional file 5.** References of all excluded full text studies.

**Additional file 6.** Risk of bias of included studies.

**Additional file 7.** Quality assessment of cost evaluation studies.

**Additional file 8.** Original reported costs / charges data.

## Data Availability

The protocol for the systematic review is available from https://www.crd.york.ac.uk/PROSPERO/display_record.php?RecordID=57592. The protocol for the systematic review was published and available from https://systematicreviewsjournal.biomedcentral.com/articles/10.1186/s13643-018-0693-x

## Abbreviations

NRCTs: Non-randomized controlled trial
RCTs: Randomized controlled trial
CBA: Controlled before-after study
ITS: Interrupted time series study
EPOC: Effective practice and organisation of care
LOS: Length of stay
WMD: Weighted mean difference
OR: Odds ratio
