# Supplementary material for: Effects of oncological care pathways in primary and secondary care on patient, professional and health systems outcomes: a systematic review and meta-analysis
Source: Syst Rev. 2020 Oct 25;9:246. doi: 10.1186/s13643-020-01498-0 (PMC7586678; doi:10.1186/s13643-020-01498-0)
Supplement: Supplementary file 1 — Additional file 1:. Overview of inclusion criteria for this systematic review. [file 13643_2020_1498_MOESM1_ESM.docx]

**Additional file 1 Overview inclusion criteria**

| Inclusion criteria:   - Participants: patients, care providers, and healthcare organizations in primary and secondary/tertiary care. - Intervention: care pathway based on four operational pathway criteria. - Comparators: cancer care pathway versus usual care, cancer care pathway versus control, cancer care pathway versus non-pathway. - Outcomes: patient, professional and system level outcome (primary) and measures regarding implementation strategies and methods (secondary). - Study designs: randomized controlled trials (RCT), non-randomized controlled trials (NRCT), controlled before-after studies (CBA), and interrupted time series studies (ITS), cost-effectiveness analyses, cost-utility analyses and cost-benefit analyses, cost analysis and comparative resource utilisation studies |
| --- |
