# Supplementary material for: Effects of oncological care pathways in primary and secondary care on patient, professional and health systems outcomes: a systematic review and meta-analysis
Source: Syst Rev. 2020 Oct 25;9:246. doi: 10.1186/s13643-020-01498-0 (PMC7586678; doi:10.1186/s13643-020-01498-0)
Supplement: Supplementary file 2 — Additional file 2. Care pathways search strategy Ovid Medline: 1946 to July 1, 2020. [file 13643_2020_1498_MOESM2_ESM.docx]

**Care pathways search strategy Ovid Medline: 1946 to June 1, 2019 (summary)**

--------------------------------------------------------------------------------

1 exp Neoplasms/

2 exp medical oncology/ or radiation oncology/ or surgical oncology/

3 oncologists/ or radiation oncologists/

4 (cancer or oncolog$ or neoplasm?).ti,ab,kw,kf.

5 (cancer or carcinoma$ or oncolog$ or malignanc$ or neoplasia? or neoplasm?).ti,ab,kw,kf.

6 (tumo?r? adj3 (malignan$ or neoplas$)).ti,ab,kw,kf.

7 or/1-6 [Cancer]

8 Critical Pathways/

9 care plan?.ti,ab,kw,kf.

10 structured care.ti,ab,kw,kf. clinical pathway/

11 intensive management.ti,ab,kw,kf.

12 (standardi?ed adj2 (care or patient care or treatment?)).ti,ab,kw,kf.

13 (care algorithm? or treatment algorithm? or therapeutic algorithm?).ti,ab,kw,kf.

14 (care path$ or clinical path$ or critical path$ or patient care path$ or pharmacotherap$ path$ or therapeutic$ path$ or treatment? path$).ti,ab,kw,kf.

15 ((care or clinical or patient care or pharmacotherap$ or therapeutic$ or treatment?) adj2 pathway?).ti,ab,kw,kf.

16 ((care or treatment) adj2 (map or maps)).ti,ab,kw,kf.

17 process map$.ti,ab,kw,kf.

18 or/8-17 [Critical Pathways]

19 7 and 18 [Cancer & Pathways]

20 ((guideline or guidelines) adj4 (local$ or adapt$ or customi$ or tailor$)).ti,ab,kw,kf.

21 ((guideline or guidelines) adj4 (pathway? or path way?)).ti,ab,kw,kf.

22 ((guideline or guidelines) adj4 (interdisciplin$ or inter-disciplin$ or crossdisciplin$ or cross disciplin$ or multidisciplin$ or multi-disciplin$ or team? or standardi?)).ti,ab,kw,kf.

23 or/20-22 [Guidelines--customized, locally adapted]

24 guideline/ or practice guideline/

25 (local or local?y or adapted or customi$ or interdisciplin$ or inter-disciplin$ or crossdisciplin$ or cross disciplin$ or multidisciplin$ or multi-disciplin$ or standardi$).ti,ab,kw,kf.

26 and/24-25 [MeSH GUideline & customization terms]

27 or/23,26 [Customized Guidelines]

28 7 and 18 [Cancer & Pathways]

29 (7 and 27) not 28 [Cancer & Customized Guidelines]

30 (randomized controlled trial or controlled clinical trial).pt. or randomized.ab. or placebo.ab. or clinical trials as topic.sh. or randomly.ab. or trial.ti.

31 exp animals/ not humans.sh.

32 30 not 31 [Cochrane RCT Filter 6.4.d Sens/Precision Maximizing]

33 intervention?.ti. or (intervention? adj6 (clinician? or collaborat$ or community or complex or DESIGN$ or doctor? or educational or family doctor? or family physician? or family practitioner? or financial or GP or general practice? or hospital? or impact? or improv$ or individuali?e? or individuali?ing or interdisciplin$ or multicomponent or multi-component or multidisciplin$ or multi-disciplin$ or multifacet$ or multi-facet$ or multimodal$ or multi-modal$ or personali?e? or personali?ing or pharmacies or pharmacist? or pharmacy or physician? or practitioner? or prescrib$ or prescription? or primary care or professional$ or provider? or regulatory or regulatory or tailor$ or target$ or team$ or usual care)).ab.

34 (pre-intervention? or preintervention? or "pre intervention?" or post-intervention? or postintervention? or "post intervention?").ti,ab.)

35 (hospital$ or patient?).hw. and (study or studies or care or health$ or practitioner? or provider? or physician? or nurse? or nursing or doctor?).ti,hw.

36 demonstration project?.ti,ab.

37 (pre-post or "pre test$" or pretest$ or posttest$ or "post test$" or (pre adj5 post)).ti,ab.

38 (pre-workshop or post-workshop or (before adj3 workshop) or (after adj3 workshop)).ti,ab.

39 trial.ti. or ((study adj3 aim?) or "our study").ab.

40 (before adj10 (after or during)).ti,ab.

41 ("quasi-experiment$" or quasiexperiment$ or "quasi random$" or quasirandom$ or "quasi control$" or quasicontrol$ or ((quasi$ or experimental) adj3 (method$ or study or trial or design$))).ti,ab,hw.

42 ("time series" adj2 interrupt$).ti,ab,hw.

43 (time points adj3 (over or multiple or three or four or five or six or seven or eight or nine or ten or eleven or twelve or month$ or hour? or day? or "more than")).ab.

44 pilot.ti.

45 Pilot projects/

46 (clinical trial or controlled clinical trial or multicenter study).pt.

47 (multicentre or multicenter or multi-centre or multi-center).ti.

48 random$.ti,ab. or controlled.ti.

49 (control adj3 (area or cohort? or compare? or condition or design or group? or intervention? or participant? or study)).ab. not (controlled clinical trial or randomized controlled trial).pt.

50 "Controlled Before-After Studies"/ or "Interrupted Time Series Analysis"/

51 evaluation studies as topic/ or prospective studies/ or retrospective studies/

52 (utili?ation or programme or programmes).ti.

53 (during adj5 period).ti,ab.

54 ((strategy or strategies) adj2 (improv$ or education$)).ti,ab.

55 "comment on".cm. or review.pt. or (review not "peer review$").ti. or randomized controlled trial.pt.

56 exp animals/ not humans.sh.

57 (animal? or beaver? or beef or bovine or breeding or bull or canine or castoris or cat or cattle or cats or chicken? or chimp$ or cow or dog or dogs or equine or foal or foals or fish or insect? horse or horses or livestock or mice or monkey? or mouse or murine or plant or plants or pork or porcine or protozoa? or purebred or rat or rats or rodent? or sheep or thoroughbred).ti. or veterinar$.ti,ab,kw,kf,hw.

58 (or/33-54) not (or/55-57) [EPOC Filter for Non-RCT designs]

59 ((systematic adj2 review) or metaanaly$ or meta=analy$ or scoping review or concept analysis).ti,kw,kf.

60 cochrane.jw.

61 meta-analysis/

62 or/59-61 [ad hoc SR/MA Filter--Cochrane Library will also be searched for related reviews]

63 (or/28-29) and (32 not 57) [RCT Results]

64 (or/28-29) and 58 [NRCT Results]

65 (or/28-29) and 62 [SR Results]

66 remove duplicates from 65 [SR results to export]

67 remove duplicates from 63 [RCT Results to export]

68 67 not 66 [RCT Results to export]

69 remove duplicates from 64

70 69 not (or/66,68) [NRCT Results to Export]
