# Supplementary material for: Effects of oncological care pathways in primary and secondary care on patient, professional and health systems outcomes: a systematic review and meta-analysis
Source: Syst Rev. 2020 Oct 25;9:246. doi: 10.1186/s13643-020-01498-0 (PMC7586678; doi:10.1186/s13643-020-01498-0)
Supplement: Supplementary file 3 — Additional file 3. Systematic review cancer care pathways PRISMA flow diagram. [file 13643_2020_1498_MOESM3_ESM.docx]

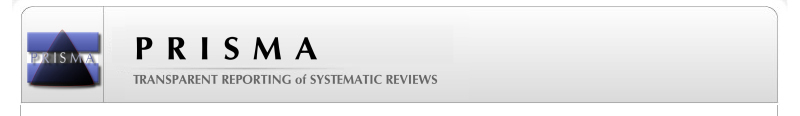
**PRISMA 2009 Flow Diagram**

Full-text articles excluded, with reasons
(n = 150)

- Study did not meet Cochrane EPOC study design inclusion criteria (reviews, case-studies, uncontrolled studies) (n = 79)
- Other intervention (comparison of other interventions than pathways, or pathway does not meet the definition) (n = 23)
- Other patient population (not cancer patients (only)) (n = 15)
- Other control (or no control group) (n = 12)
- Abstract only (n = 9)
- Protocol only (n = 6)
- Not available as full-text (n = 3)
- No information about care pathway (n = 2)
- Other outcomes (no outcomes as described in protocol) (n = 1)

Studies included in quantitative synthesis (meta-analysis)
(n = 3)

Studies included in qualitative synthesis
(n = 8)

Full-text articles assessed for eligibility
(n = 158)

Records excluded
(n = 13,689)

Records screened
(n = 13,847)

Records after duplicates removed
(n = 13,847)

Additional records identified through other sources
(n = 241)

## Identification

## Eligibility

## Included

## Screening

Records identified through database searching
(n = 13,629)
