# Supplementary material for: Effects of oncological care pathways in primary and secondary care on patient, professional and health systems outcomes: a systematic review and meta-analysis
Source: Syst Rev. 2020 Oct 25;9:246. doi: 10.1186/s13643-020-01498-0 (PMC7586678; doi:10.1186/s13643-020-01498-0)
Supplement: Supplementary file 4 — Additional file 4. Excluded full text studies with the reason for exclusion. [file 13643_2020_1498_MOESM4_ESM.docx]

**Additional file 4 Excluded studies: reason for exclusion**

|  | **Study ID** | **Reason for exclusion** | **Patients/subjects** | **Study design** | **Country** | **Outcomes** |
| --- | --- | --- | --- | --- | --- | --- |
| 1 | Abou-Haidar H, et al. 2014 | Study did not meet Cochrane EPOC study design criteria | prostate cancer | retrospective study | Canada | Duration of hospital stay, complications, post-discharge emergency department visits and readmissions |
| 2 | Adams J. 2000. | Unavailable full-text |  |  |  |  |
| 3 | Afonso A, et al. 2017 | Not cancer patients | patients undergoing microsurgical breast reconstruction (not cancer patients) | retrospective cohort study | USA | Hospital length of stay and total postoperative opioid consumption |
| 4 | Aggarwal R, et al. 2014 | Study did not meet Cochrane EPOC study design criteria | esophageal cancer surgery | systematic review | NA | Pre-, intra-, and post-operative measures: epidural analgesia, early catheter and tube removal, and early post-operative mobilization |
| 5 | Andtbacka RH, et al. 2006 | No control | breast cancer surgery | retrospective and rospective cohort study? | USA | Postoperative venous thromboembolism (VTE) within 60 days |
| 6 | Baade PD, et al. 2010 | Protocol only | prostate cancer | longitudinal epidemiological study without a control group | Australia | Demographics, medical history, patterns of care, disease and treatment characteristics, quality of life and psychological adjustment, detailed treatment information, road travel distances from patients’ residences to treatment centres |
| 7 | Bae HW, et al. 2017 | Study did not meet Cochrane EPOC study design criteria | colorectal cancer patient | retrospective study | Korea | Length of hospital stay, incidence of postoperative complication, the value of preoperative prognostic nutritional index (PNI), disease free survival (DFS), overall survival (OS) |
| 8 | Baffert S, et al. 2015 | Protocol only | breast cancer | observational, prospective, multicenter study | France | Cost of pathway, patient satisfaction and work reintegration, quality, coordination and access to innovation |
| 9 | Bakens MJAM, et al. 2016 | Abstract only | pancreatic cancer | retrospective study, with before and after pathway groups |  | Unclear |
| 10 | Bao H, et al. 2016 | No information about CP | Breast, colon and rectal cancer | Interrupted time series study | China | Length of hospital stay (median, IQR), complications, quality indicators about the compliance with the care process |
| 11 | Barrett J, et al. 2008 | Study did not meet Cochrane EPOC study design criteria | lung cancer | retrospective case-control study | UK | Referral and time to diagnosis |
| 12 | Barrett J, et al. 2006 | Study did not meet Cochrane EPOC study design criteria | colorectal cancer | observation study | UK | 7 clinical features: diarrhoea, constipation, rectal bleeding, abdominal pain, the finding of an abdominal or rectal mass on examination, anaemia, positive faecal occult blood tests. And time intervals: first consultation to first referral, first referral to diagnosis |
| 13 | Barrett J, et al. 2010 | Study did not meet Cochrane EPOC study design criteria | ovarian cancer | cohort study | UK | Patients taking specific routes to diagnosis, together with the time taken to diagnosis |
| 14 | Bosch C, et al. 2010 | Abstract only | breast cancer | ?? | Germany | Hospitalization |
| 15 | Brannstrom M, et al. 2016 | Not cancer patients only | patients in the last days of their lives | exploratory, controlled before-after study | Sweden | Patients' symptoms: distress and well being during the last days of life |
| 16 | Chan RJ, et al. 2016 | Study did not meet Cochrane EPOC study design criteria | patients in the last days of their lives | systematic review | NA | Primairy outcomes: physical symptom severity, psychological symptom severity, harms. Secondary outcomes: advanced care planning, communication between healthcare teams and families, carers’ well-being, grief and bereavement, patient/staff/carers’ satisfaction, staff confidence, cost of intervention, cost of care, medication/treatment use, spiritual needs |
| 17 | Chang PL, et al. 2002 | Other intervention | renal cell carcinoma | ?? | Taiwan/UK | Length of stay, admission charges, six quality indicators and the advantages of the web based pathway. |
| 18 | Chang PL, et al. 2000 | Study did not meet Cochrane EPOC study design criteria | renal cell carcinoma | retrospective cohort study | Taiwan | Length of stay, average admission charges, and 8 quality indicators |
| 19 | Chang SS, et al. 2002 | Not cancer patients | radical cystectomy (no oncology patients) | ?? | USA | Complication rate, postoperative ileus, blood los, mortality rate |
| 20 | Chang SS, et al. 2002 | Study did not meet Cochrane EPOC study design criteria | germ cell carcinoma | retrospectieve study | USA | Clinical/pathological stage, hospital stay, postoperative care and complications rate |
| 21 | Chang WC, et al. 2003 | Not cancer patients only | patients who recieved laparoscopy assisted vaginal hysterectomy (mostly patients with myoma uteri) | retrospective study | Taiwan | Delay of operation, blood transfusion, duartion of post-operative antibiotics, patient mortality, complications, readmissions, length of stay, duration of operation, duration of anesthesia |
| 22 | Chang WC, et al. 2003 | Not cancer patients only | most patients with myoma uturi (a few patients with cancer) | retrospective study | Taiwan | Costs, operation time, length of stay |
| 23 | Chase DM, et al. 2008 | Study did not meet Cochrane EPOC study design criteria | gynaecologic oncology patients | retrospective study (without control group) | USA | Readmissions, mortality, complication rates |
| 24 | Choi JW, et al. 2013 | Other intervention | gastric cancer | retrospective study | Korea | Discharge, readmission, complications |
| 25 | Choi JY. 2011 | Study did not meet Cochrane EPOC study design criteria | hepatocelluar carcinoma | descriptive article about treatment algorithm | Korea |  |
| 26 | Cohen J, et al. 1997 | Other intervention | head and neck cancer | before-after trial | USA | Length of stay, costs of hospitalization, and variance tracking |
| 27 | Costantini M, et al. 2011 | Protocol only |  | Randomized cluster trial | Italy | Overall quality of life |
| 28 | Costantini M, et al. 2014 | Other outcomes | cancer patients | before after cluster phase II trials | Italy | Outcomes reported by family members |
| 29 | Costantini M, et al. 2014 | Does not meet CP definition | cancer patients | pragmatic cluster randomised trial | Italy | Overall quality of life |
| 30 | Dautremont JF, et al. 2016 | Study did not meet Cochrane EPOC study design criteria | Head and neck oncologic surgery | before-after study | Canada | Emergency department visits, outpatient visits, inpatients admissions, physician claims, post discharge costs: impatient costs, outpatients costs, emergency department costs, medical doctor claims costs |
| 31 | Dautremont JF, et al 2013 | Study did not meet Cochrane EPOC study design criteria | Head and neck surgery | before-after study | Canada | Length of stay, return to operation room, readmission to Intensive Care Unit, time to decannulation, pneumonia |
| 32 | de Luc K. 2000 | Study did not meet Cochrane EPOC study design criteria | breast disease patients (including cancer patients as subgroup) | quasi experimental case study | UK | Waiting times, diagnostic procedures performed. For cancer patients: discussion in multidisciplinary meeting, seeing the specialist breast nurse, first seeing specialist breast nurse in the outpatients, number of times patients seeing specialist breast nurse. |
| 33 | Delgado A, et al. 2013 | No control | oncology patients | unclear | USA | Unclear |
| 34 | Dyrop HB, et al. 2013 | Study did not meet Cochrane EPOC study design criteria | bone and soft tissue sarcomas | retrospective study | Denmark | Milestones, time intervals, performed diagnostics, and tumor size |
| 35 | Fackler-Schwalbe I, et al. 2014 | Abstract only |  | unclear | Germany | Unclear |
| 36 | Fields KK, et al. 2017 | Other intervention |  | adherence to pathways |  | Unclear |
| 37 | Fiore JF, et al. 2016 | Study did not meet Cochrane EPOC study design criteria | patients undergoing elective lung resection | systematic review | NA | LOS (ie, time from surgery until first discharge), postoperative complications, readmissions, mortality rates, and patient-reported outcomes (PROs) |
| 38 | Forrest LF, et al. 2017 | Study did not meet Cochrane EPOC study design criteria | lung cancer | systematic review and meta-analysis | NA | Stage at diagnosis or the length of time spent within an interval on the care pathway, socio-economic position |
| 39 | Gasbarrini A, et al. 2010 | Other intervention | spinal metastases. | semiprospective clinical study | Italy | Neurologic function, local recurrence/development rate comparison, postoperative survival time |
| 40 | Gerardi MA, et al. 2008 | Does not meet CP definition | advanced ovarian and primary peritoneal cancers | case–control methodology | USA | The median time to flatus, median time to tolerance of diet, length of hospital stay, 30-day post-operative hospital cost, 30-day readmission rate, hospital cost |
| 41 | Gordon SA, et al. 2016 | Study did not meet Cochrane EPOC study design criteria | head and neck cancer surgery | a systematic review | NA | Setting, length of stay, complications, readmission, costs, and case length |
| 42 | Grau C, et al. 2011. | Study did not meet Cochrane EPOC study design criteria | patients with squamous cell carcinoma of the oral cavity, pharynx, and larynx | retrospective study | Denmark | Total time from first healthcare contact to start of definitive treatment, median time used for diagnosis, median time from diagnosis to treatment start |
| 43 | Gustafsson UO et al. 2011 | Study did not meet Cochrane EPOC study design criteria | colorectal cancer surgery | before-after study | Sweden | Postoperative symptoms, complications, length of stay, 30-day morbidity, mortality |
| 44 | Hingh IDE, et al. 2014 | Study did not meet Cochrane EPOC study design criteria | pancreatic cancer | retrospective study | Netherlands | Proportion of patients without pre-operative biliary drainage, duration of referral to surgery, duration of 1st outpatient visit to final treatment and the number of preoperative outpatient visits prior to treatment |
| 45 | Hirasaki S, et al. 2004 | Study did not meet Cochrane EPOC study design criteria | mucosal gastric carcinoma | retrospective study | Japan | Total length of hospitalization, preoperative stay, postoperative stay, hospital costs, total costs, the number of endoscopic examination, operation times |
| 46 | Hoverman JR, et al. 2011 | Study did not meet Cochrane EPOC study design criteria | colon cancer | retrospective cohort design | USA | Disease-free survival in patients receiving adjuvant treatment and overall survival and costs |
| 47 | Hsu YC, et al. 2008 | Study did not meet Cochrane EPOC study design criteria | localized prostate cancer | retrospective study? | Taiwan | Pathway variations and length of hospital stay |
| 48 | Hubner M, et al. 2015 | Study did not meet Cochrane EPOC study design criteria | colorectal surgery | a retrospective cohort study | Switzerland | Return of bowel function (flatus/stool), postoperative complications, length of stay, total hospital stay, number of preoperative days, early readmissions within 30 days after surgery |
| 49 | Husbands JM, et al. 1999 | Study did not meet Cochrane EPOC study design criteria | head and neck oncologic surgery | before-after study | USA | Median charge per patient, length of stay, readmission rate, complication rate, mortality rate |
| 50 | Iijima K, et al. 2003 | Study did not meet Cochrane EPOC study design criteria | patients with neoplastic diseases | retrospective study | Japan | Differences in procedures and therapy between the pathway group and control group, postoperative complications, readmissions, or overall deaths for one year |
| 51 | Ise Y, et al. 2003 | Other intervention | stomach cancer | cost-effectiveness analysis. | Japan | Pre- and postoperative stay at the hospital, length of stay, costs |
| 52 | Ishiguro S, et al. 2008 | Study did not meet Cochrane EPOC study design criteria | colorectal cancer | retrospective study | Japan | Hospital stay, mortality, morbidity, reoperation, readmission |
| 53 | Jackman DM, et al. 2017 | Study did not meet Cochrane EPOC study design criteria | lung cancer | before-after study | USA | Costs of care & clinical outcomes (survival times) |
| 54 | Jakobsen JK, et al. 2016 | Study did not meet Cochrane EPOC study design criteria | penile cancer | observational study | Denmark | Median time intervals (in calendar days) with interquartile range were the main outcome measure |
| 55 | Jensen KH, et al. 2015 | Study did not meet Cochrane EPOC study design criteria | colorectal cancer | retrospective cohort study | Denmark | Median referral time from GP to endoscopy, time from referral to oncological treatment, long-term survival rate |
| 56 | Kagan SH, et al. 2002 | No control | head and neck surgery | retrospective study? | USA | LOS and age |
| 57 | Kardos SV, et al. 2016 | Abstract only | bladder cancer | unclear | USA | LOS, complications, readmissions |
| 58 | Katterhagen G. 1996 | Unavailable full-text |  |  |  |  |
| 59 | Keetch DW, et al. 1998 | Other control | adenocarcinoma of the prostate | prospective study | USA | Hospital stay, hospital costs |
| 60 | Kennedy EP, et al. 2009 | Other patient population | patients underwent distal pancreatectomy (44% malignant in prepathway group and 56% in postpathway group) | retrospective study | USA | Operative length, postoperative complications, Perioperative mortality, length of postoperative hospital stay, readmission rate, hospital costs |
| 61 | Khan SA, et al. 2013 | Study did not meet Cochrane EPOC study design criteria | patients undergoing elective colorectal surgery (83% colorectal cancer in laparascopic group, 76% in the open-ERAS group) | prospective observational study | UK | Length of stay and quality of life (HQoL) |
| 62 | Khoo CK, et al. 2007 | Other intervention | patients undergoing elective resection for colorectal cancer | prospective randomized controlled trial (RCT) | UK | Postoperative stay, and achievement of independence milestones. Secondary endpoints were postoperative complications, readmission rates, and mortality. |
| 63 | Kim HS, et al. 2015 | Other intervention | gastric cancer | retrospective study | South Korea | Unclear |
| 64 | Kiyama T, et al. 2003 | Study did not meet Cochrane EPOC study design criteria | gastrectomy patients | economic analysis | Japan | Length of hospital stay, costs of hospital stay: cost per bed |
| 65 | Klinkhammer-Schalke M, et al. 2012 | Other intervention | breast cancer | randomised controlled trial (RCT) | Germany & UK | ‘diseased’ QoL (any QoL measure below 50 points) using a QoL profile |
| 66 | Klinkhammer-Schalke M, et al. 2015 | Protocol only | colorectal cancer patients | RCT | Germany | Quality of life |
| 67 | Kobayashi ST, et al. 2017 | Study did not meet Cochrane EPOC study design criteria | rectal cancer | a retrospective study | Brazil | The interval between the first medical consultation date and postoperative day 30. The resources considered: consultations (medical, multidisciplinary, dental), computed tomography (CT) scans, MRI scans, colonoscopies, emergency room care, the chemotherapy regimen, radiotherapy sessions, and the surgery, as well as hospital stays, ICU stays, day hospital stays, and costs. |
| 68 | Konety BR, et al. 1996 | Study did not meet Cochrane EPOC study design criteria | radical retropubic prostatectomy | before-after study | USA | Hospital charges, length of stay, operating room time, units of packed red cells transfused, morbidity, mortality |
| 69 | Kreys ED, et al. 2013 | Study did not meet Cochrane EPOC study design criteria | breast, lung, or colorectal cancer treatment | a retrospective single-group, pre-test–post-test design. | USA | Compliance rate for chemotherapy, supportive care site compliance, patient drug costs, hospitalization costs |
| 70 | Langham J, et al. 2013 | Study did not meet Cochrane EPOC study design criteria | diagnoses such as cancer | ?? | UK | Unclear |
| 71 | Lee L, et al. 2013 | Study did not meet Cochrane EPOC study design criteria | elective oesophagectomy | before-after study | Canada | Length of hospital stay, complication rates, readmission within 30 days of surgery, in-hospital mortality rate, costs (pathway dependent & total) |
| 72 | Leibman BD, et al. 1998 | Does not meet CP definition | prostate cancer | retrospective study? | USA | Total hospital charges and length of stay, physician volume, patient satisfaction, Incidence of postoperative urinary tract infections, atelectesis, wound infection, and paralytic ileus |
| 73 | Lemmens L, et al. 2009 | Study did not meet Cochrane EPOC study design criteria | patient undergoing oncology surgery of the gastrointestinal tract | systematic review | NA | Postoperative outcome measures |
| 74 | Letton C, et al. 2013 | Abstract only | gynaecological oncology | unclear | UK | Duration of intraperitoneal drains, urethral catheters, intravenous fluids postoperatively, time to eat and drink, time to mobilization, length of hospital stay |
| 75 | Lindberg P, et al. 2016 | Other intervention | colorectal cancer patients | RCT | Germany | Quality of life |
| 76 | Llobera J, et al. 2017 | Protocol only | palliative care patients (oncology and non-oncology) | RCT | Spain | Early identification, evaluation of case complexity, level of case complexity, use and cost of hospital and primary care services, and quality of life during the last month of life |
| 77 | Lorenz W, et al. 2005 | Abstract only | breast cancer | prospective trial | Germany | Unclear |
| 78 | Lyhne NM, et al. 2013 | Study did not meet Cochrane EPOC study design criteria | head and neck cancer: patients with squamous cell carcinoma of the oral cavity, pharynx and larynx | retrospective study? | Denmark | Time intervals: Time from first healthcare contact with a cancer suspicion until final histopathological diagnosis was presented to the patient; Time from diagnosis to first day of either RT or definitive surgery; Time from suspicion of cancer to start of definitive treatment. Number of diagnostic imaging procedures performed (ultra sound (US), CT, magnetic resonance imaging (MRI) and (MRI) and positron emission tomography (PET)-CT). |
| 79 | Markar SR, et al. 2015 | Study did not meet Cochrane EPOC study design criteria | oesophagus cancer | systematic review | NA | Incidence of postoperative mortality, anastomotic leak and pulmonary complications, and secondary outcomes were length of hospital stay and the incidence of 30-day readmission. |
| 80 | Markar SR, et al. 2014 | No control | oesophagus cancer | prospective study? | USA & UK | Use of neo-adjuvant therapy, blood loss, intraoperative fluid administration, ICU stay, length of hospital stay, post operative complications |
| 81 | Maruyama R, et al. 2006 | Study did not meet Cochrane EPOC study design criteria | lung cancer, metastatic lung cancer, or a nodule that was suspected to be malignant | retrospective study | Japan | Length of hospitalization, hospital charges, and the outcome for video-assisted thoracoscopic pulmonary resection (VATPR) |
| 82 | Mashhad University of Medical S. 2014 | Protocol only | colorectal cancer patients | prospective randomized trial | Iran | Length of stay, postoperative complications and patient satisfaction |
| 83 | McCray DK, et al. 2017 | Study did not meet Cochrane EPOC study design criteria | breast cancer | retrospective review | USA | Pre-operative MRI utilization |
| 84 | Mori T, et al. 2017 | Study did not meet Cochrane EPOC study design criteria | lung cancer | retrospective study | Japan | Mean duration of the pathway, mean distance between patients’ home and our hospital |
| 85 | Muehling BM, et al. 2008 | No information about CP | Fast track lung resections | Prospective randomized controlled pilot study | Germany | Complications (pulmonary, cardiovascular, technical), reoperation, morbidity, mortality, length of stay at the ICU (median), day of discharge (median) |
| 86 | Munitiz V, et al. 2010 | Study did not meet Cochrane EPOC study design criteria | oesophageal neoplasia | retrospective study | Spain | Morbidity rates, pulmonary complications, length of hospital stay |
| 87 | Neo PS, et al. 2012 | Does not meet CP definition | patients in oncology ward | retrospective study? | Singapore | Five types of end-of-life symptoms |
| 88 | Nunez Mora C, et al. 2001 | Study did not meet Cochrane EPOC study design criteria | bladder tumors | retrospective study | Spain | Duration of hospital stay |
| 89 | Nussbaum DP, et al. 2014 | Other patient population | patients undergoing distal pancreatectomy (DP) of which 26% in each groups Pancreatic cancer | retrospective review | USA | Complication rates, 30-d resurgery, readmissions |
| 90 | O'Connell DA, et al. 2015 | Does not meet CP definition | patients undergoing major head and neck cancer resections for cancer treatment | retrospective case-control study | Canada | Length of hospital stay, complications (minor and major) |
| 91 | Ogawa T, et al. 2004 | Study did not meet Cochrane EPOC study design criteria | patients with thyroid and parotid tumors | retrospective review | Japan | Clinical outcomes, length of hospitalization and costs |
| 92 | Oh JK, et al. 2014 | Study did not meet Cochrane EPOC study design criteria | uterine cervix cancer | retrospective review | Korea | Overall and postoperative hospital stay, catheter time. In questionnaire for the ward nurses: their perception on patient’s satisfaction, subjective benefits and relative incidence of complications. |
| 93 | Ortega-Lucea SM, et al. 2015 | Does not meet CP definition | patients with localized muscle-invasive bladder cancer | ambispective cohort study | spain | Intraoperative variables, postoperative complications (according to the Clavien classification), hospital stay and recovery stay. |
| 94 | Page BJ, et al. 2013 | Other intervention | lung cancer | retrospective study | Australia | Interval times: (A) from receipt of referral to first specialist appointment (FSA), (B) FSA to first pathological (cytology or histology) diagnosis (FPD), (C) FPD to first multidisiciplinary team discussion (MDT) and (D) MDT to first definitive treatment (FDT). |
| 95 | Pease NJ, et al. 2004 | Study did not meet Cochrane EPOC study design criteria | patients with suspected malignant spinal cord compression | two retrospective audits | UK | Early mobilisation, complication rate, patient survival, neurological function |
| 96 | Pellino G, et al. 2017 | Study did not meet Cochrane EPOC study design criteria | colorectal cancer | systematic review | NA | Primary outcomes: symptoms of colorectal cancer in pregnancy (CRC-p), and the outcome of the mother and foetus according to CRC features and location, gestational age and treatment delivered. Secondary outcomes: incidence of CRC-p, exposure to chemotherapy in utero and the development of a treatment algorithm suitable for these patients. |
| 97 | Pengfei LUO, et al. 2015 | Study did not meet Cochrane EPOC study design criteria | liver cancer | unclear | China | Time to functional recovery, postoperative length of stay, postoperative complications, hospital costs, time to mobility, time to first passage of stool, readmission and mortality. |
| 98 | Peters M, et al. 2016 | Abstract only | cancer care pathways | systematic review | NA | Effective implementation, sustainability, and evaluation of cancer care pathways. |
| 99 | Pfau PR, et al. 2004 | Not cancer patients only | patients with acute upper nonvariceal GI hemorrhage (probably not only cancer patients) | prospective study? | USA | Length of stay, time to endoscopy, utilization of potentially unnecessary radiological tests, acid suppression, and cost of care |
| 100 | Pirnejad H, et al. 2013 | Other intervention | cancer patients | retrospective study | Netherlands | Turn-around-time 109 Paclitaxel–Carboplatin chemotherapy courses. The chemotherapy-postponing rate, administration record completion rate, and admission rate of day care unit before and after the implementation. |
| 101 | Porteous GH, et al. 2015 | Study did not meet Cochrane EPOC study design criteria | esophageal cancer | retrospective study | USA | Intensive care unit and hospital length of stay, surgical complications, and morbidity and mortality rates. |
| 102 | Porter GA, et al. 2000 | Not cancer patients only | patients undergoing elective pancreaticoduodenectomy (PD), mostly patients with carcinoma, but a few with pancreatis or other diseases |  |  | Perioperative mortality, postoperative morbidity, length of stay, readmissions, and postoperative clinic visits. |
| 103 | Preston SR, et al. 2013 | Does not meet CP definition | oesophageal cancer | unclear | USA | Intraoperative blood loss, fluid administration and duration of surgery. Postoperative outcomes: length of hospital and critical care stay, and postoperative complications. |
| 104 | Quraishi T, et al. 2012 | Abstract only | uro-oncology surgical services | unclear | UK | LOS, mortality, morbidity, readmissions within 30 days |
| 105 | Raman V, et al. 2016 | Study did not meet Cochrane EPOC study design criteria | esophagectomy for patients with cancer | retrospective review | USA | Mortality, major complications, hospitalization, and total charges |
| 106 | Rashid OM, et al. 2016 | Study did not meet Cochrane EPOC study design criteria | patients with borderline resectable pancreatic adenocarcinoma | retrospective study | USA | Pancreatectomy rates, margin status, pathologic response, disease-free survival (DFS), disease-specific survival (DSS), and overall survival (OS) |
| 107 | Rich-Ruiz M, et al. 2006 | Other patient population | patients undergoing surgery for benign prostatic hyperplasia or cancer of the bladder through transurethral resection | randomized study | Spain | Length of hospital stay, complication rate, patient satisfaction |
| 108 | Richter-Ehrenstein C, et al. 2012 | No control | breast surgery patients with benign and malignant disease | unclear | Germany | Hospital stay, total costs per case, readmission rate, and patients’ satisfaction. |
| 109 | Roberts HC, et al. 2004 | Not cancer patients | patients aged 65 years and over with a femoral neck fracture (no cancer) | prospective controlled before and after study | UK | Primary outcome: length of stay on the orthopaedic unit. Secondary outcomes: ambulation at discharge, discharge destination, in-hospital complications, 30 day mortality, readmission within 30 days of discharge, postoperative days the patient sat out of bed and walked. |
| 110 | Sancho C, et al. 2010 | No control | rectal cancer (RC) treatment | unclear | Spain | Imaging methods for staging, neoadjuvant therapy, surgical treatment and pathology, and use of resources. |
| 111 | Santillan A, et al. 2008 | Other intervention | gynecologic cancers | retrospective and prospective cohort study? | USA | PAP test rate and costs |
| 112 | Santoso U, et al. 2002 | Study did not meet Cochrane EPOC study design criteria | breast cancer | before-after study | Singapore | Length of stay, total hospital costs, postoperative complications, unscheduled readmissions within 15 days of surgery |
| 113 | Schwarzbach M, et al. 2010 | Other patient population | patients undergoing lobectomy: (15,7% in CP group and 4,7% in prepathway group had benign tumors) | unclear | Germany | Duration of catheter placement, pain intensity, respiratory exercising, and mobilization. Outcome quality: morbidity, mortality, reoperations, readmissions, and cost of hospital stay |
| 114 | Schwarzbach MH, et al. 2010 | Other patient population | patients undergoing video-assisted thoracoscopy (VATS) (more or less 40% malignant) | unclear | Germany | Duration of catheter placement, pain intensity, respiratory exercising, and mobilization. Outcome quality: morbidity, mortality, reoperations, readmissions, and cost of hospital stay |
| 115 | Scott JA, et al. 2013 | Abstract only | patients with breast, colon and lung cancer | retrospective study? | ?? | Sum of allowed cancer costs 270 days after chemotherapy, and probability of inpatient admission |
| 116 | Seo HS, et al. 2012 | No control | gastric cancer surgery | prospective study? | Korea | Length of stay, complication rate, total cost paid at the patient’s discharge, and the daily hospital income |
| 117 | Shamji FM, et al. 2013 | Study did not meet Cochrane EPOC study design criteria | lung cancer | observational? | Canada | Time intervals |
| 118 | Sherman D, et al. 2001 | Abstract only | larynx and hypopharynx cancer | retrospective study | Canada | Length of postoperative inpatient stay, readmissions, complications |
| 119 | Shetiwy M, et al. 2017 | Does not meet CP definition | colorectal cancer | controlled clinical trial | Egypt | Length of hospital stay. Secondary outcomes: times of removal of nasogastric tubes (NGTs), successful enteral feeding, and removal of drains, postoperative complications, intra-hospital mortality, and rate of readmission. |
| 120 | Sivakumaran Y, et al. 2013 | Study did not meet Cochrane EPOC study design criteria | colorectal cancer | retrospective observational cohort study | Australia | (A) mode of presentation: emergency versus elective (B) key time intervals and (C) performance against recommended guidelines |
| 121 | So JB, et al. 2008 | Does not meet CP definition | gastric cancer | retrospective study | Singapore | Postoperative length of hospital stay, hospital costs |
| 122 | Song XP, et al. 2014 | Study did not meet Cochrane EPOC study design criteria | gastrointestinal Cancer | meta-analysis | NA | Length of stay, costs and patient satisfaction |
| 123 | Soria-Aledo V, et al. 2011 | Does not meet CP definition | colorectal Cancer | ambispective presurgery and postsurgery study | Spain | Postoperative stay, compliance with antibiotic prophylaxis, compliance with the staging study, mortality, rate of infection, and reoperations. |
| 124 | Stephen AE, et al. 2003 | Not cancer patients only | colorecctal cancer (83% in pre group and 67% in post group) and diverticular disease | retrospective study | USA | Length of stay, postoperative complications, readmissions, and cost per patient |
| 125 | Stocker R, et al. 2013 | Study did not meet Cochrane EPOC study design criteria | non cancer and cancer | systematic review | NA | Number of patients placed on the LCP, length of time patients spent on the LCP |
| 126 | Takagi J. 1996 | Unavailable full-text |  |  |  |  |
| 127 | Tateno Y, et al. 2012 | Study did not meet Cochrane EPOC study design criteria | cancer patient (different cancers) | retrospective study | Japan | Pain management |
| 128 | Tomaszek SC, et al. 2010 | Study did not meet Cochrane EPOC study design criteria | oesophageal cancer or Barrett’s oesophagus with high-grade dysplasia | retrospective study | USA | anastomotic leak rates, length of hospitalisation, re-admission and other complications. |
| 129 | Tomiki Y, et al. 2015 | Study did not meet Cochrane EPOC study design criteria | colorectal neoplasms | retrospective study | Japan | Hospital stay |
| 130 | Turini IG, et al. 2016 | Study did not meet Cochrane EPOC study design criteria | patients who underwent Robot Assisted Laparoscopic Radical Prostatectomy (RALRP) | retrospective chart review | ?? | Clinical patient care outcomes, quality of life and satisfaction |
| 131 | Una E, et al. 2010 | Study did not meet Cochrane EPOC study design criteria | Rectal Cancer | retrospective cross-sectional study | Spain | Time intervals, patient satisfaction |
| 132 | Valentin-Lopez B, et al. 2012 | Study did not meet Cochrane EPOC study design criteria | colorectal cancer | retrospective study and prospective data collection of pathway group | Spain | Referral criteria met, waiting times, number of cancers diagnosed, endoscopic polypectomies performed, surgery for CRC and the stage of disease at surgery. |
| 133 | Vallverdu-Cartie H, et al. 2011 | Study did not meet Cochrane EPOC study design criteria | colorectal cancer | longitudinal and observational study | Spain | Origin of patient’s referral, date of entry in FTDP, date of first hospital visit, date of colonoscopy, date of colorectal surgeon consultation and date of surgical intervention |
| 134 | Van Beek K, et al. 2016 | Study did not meet Cochrane EPOC study design criteria | cancer patients | systematic literature review | NA | ICP criteria |
| 135 | van Dam PA, et al. 2013 | No control | breast cancer | prospective systematic evaluation | Belgium | Clinical outcome measures, service indicators, team indicators, process indicators and financial indicators: treatment according the guidelines, patient satisfaction, progression free 4-year survival, overall 4-year survival |
| 136 | van Hoeve J, et al. 2014 | Study did not meet Cochrane EPOC study design criteria | breast cancer | retrospective study | Netherlands | Clinical outcomes and time intervals |
| 137 | van Hoeve JC, et al. 2015 | Study did not meet Cochrane EPOC study design criteria | rectal cancer | retrospective study | Netherlands | Clinical outcomes and time intervals |
| 138 | Van Houdt S, et al. 2013 | Study did not meet Cochrane EPOC study design criteria | radical prostatectomy patients (cancer patients?) | exploratory trial | Belgium | Quality of care was translated into process and outcome indicators. Quality of care was translated into patient-perceived quality indicators. And the Distress thermometer. |
| 139 | van Zelm R,et al. 2017 | Study did not meet Cochrane EPOC study design criteria | colorectal cancer surgery | systematic search | NA | Key interventions |
| 140 | Varela-Centelles P, et al. 2017 | Study did not meet Cochrane EPOC study design criteria | oral and oropharyngeal cancer patients | quantitative systematic review | NA | Five measures (patient, primary care, diagnosis, total diagnosis and total treatment intervals). |
| 141 | Verhofstede R, et al. 2012 | Study did not meet Cochrane EPOC study design criteria | patients dying in an acute geriatric ward | study protocol | Belgium | Symptom frequency and symptom burden of patients in the last 48 hours of life |
| 142 | Wang ML, et al. 2017 | Not cancer patients | colorectal polyps | retrospective study | China | The hospitalization cost, medicine cost, hospitalization time, satisfaction |
| 143 | Wu CL, et al. 2015 | No control | colorectal surgery patients | unclear |  | Length of stay and patient satisfaction |
| 144 | Xuping S, et al. 2014 | Study did not meet Cochrane EPOC study design criteria | patients with the first diagnosis as uterine fibroids | meta-analysis | NA | Length of stay, days of waiting for surgery, inpatient expenditures |
| 145 | Yetzer JG, et al. 2017 | Study did not meet Cochrane EPOC study design criteria | patiens undergoing maxillofacial head and neck surgery |  | USA | Length-of-stay metrics, infection rates, transfers to the intensive care unit, and unplanned return to the operating room. |
| 146 | Yeung JK, et al. 2014 | Study did not meet Cochrane EPOC study design criteria | head and nech cancer | before-after study | Canada | Pulmonary complications, hospital length of stay, rate of flap reoperation |
| 147 | Yueh B, et al. 2003 | Other control | head and neck cancer | cohort study? | USA | LOS after laryngectomy |
| 148 | Zauner G, et al. 2014 | Study did not meet Cochrane EPOC study design criteria | lung cancer | exploratorive study | Austria |  |
| 149 | Zhang Y, et al. 2017 | Other control | lung cancer | cost and survival analysis | USA | Clinical outcomes: survival, and costs |
| 150 | Zhu L, et al. 2014 | Study did not meet Cochrane EPOC study design criteria | hepatocellular carcinoma | retrospective study and prospective data collection of pathway group | China | Hospital costs, LOHS and early clinical outcomes |

CP: Care Pathway; NA: Not Applicable.
