# Supplementary material for: Effects of oncological care pathways in primary and secondary care on patient, professional and health systems outcomes: a systematic review and meta-analysis
Source: Syst Rev. 2020 Oct 25;9:246. doi: 10.1186/s13643-020-01498-0 (PMC7586678; doi:10.1186/s13643-020-01498-0)
Supplement: Supplementary file 5 — Additional file 5. References of all excluded full text studies. [file 13643_2020_1498_MOESM5_ESM.docx]

**Additional file 5 References excluded studies**

Abou-Haidar, H., Abourbih, S., Braganza, D., Qaoud, T.A., Lee, L., Carli, F., … Kassouf, W. (2014). Enhanced recovery pathway for radical prostatectomy: Implementation and evaluation in a universal healthcare system. *Can Urol Assoc J, 8*(11-12), 418−23.

Adams, J.(2000). Clinical care pathways: decreasing resource utilization in head and neck surgical patients. *ORL Head Neck Nurs, 18*(2), 23.

Afonso, A., Oskar, S., Tan, K.S., Disa, J.J., Mehrara, B.J., Ceyhan, J., & Dayan, J.H. (2017). Is Enhanced Recovery the New Standard of Care in Microsurgical Breast Reconstruction? *Plast Reconstr Surg, 139*(5), 1053−61.

Aggarwal, R., Buchholz, J., & Miyasaka, K. (2014). A systematic review of clinical care pathways for esophageal cancer surgery. *Diseases of the Esophagus, 27* S1, 76A.

Andtbacka, R.H., Babiera, G., Singletary, S.E., Hunt, K.K., Meric-Bernstam, F., Feig, B.W., … Kuerer, H.M. (2006). Incidence and prevention of venous thromboembolism in patients undergoing breast cancer surgery and treated according to clinical pathways. *Ann Surg, 243*(1), 96−101.

Baade, P.D., Aitken, J.F., Ferguson, M., Gardiner, R.A., & Chambers, S.K. (2010). Diagnostic and treatment pathways for men with prostate cancer in Queensland: investigating spatial and demographic inequalities. *BMC Cancer, 10*, 452.

Bae, H.W., Han, J., & Min, B.S. (2017). Evaluation of a critical pathway application to improve colorectal cancer outcomes: A propensity scoring matching analysis. *Surgical Endoscopy and Other Interventional Techniques, 31*, S181.

Baffert, S., Hoang, H.L., Bredart, A., Asselain, B., Alran, S., Berseneff, H., … Héquet, D. (2015). The patient-breast cancer care pathway: how could it be optimized? *BMC Cancer, 15*, 394.

Bakens, M.J.A.M., Bongers, M., Demelinne, J., Lemmens, V.E.P.P., & De Hingh, I.H.J.T. (2016). Implementation of a preoperative care pathway for patients with suspected periampullary cancer: Higher quality of care in less time. *Hpb, 18*, e750.

Bao H, Yang F, Su S, Wang X, Zhang M, Xiao Y, … Liu, M. (2026). Evaluating the effect of clinical care pathways on quality of cancer care: analysis of breast, colon and rectal cancer pathways. *Journal of Cancer Research and Clinical Oncology, 142*(5), 1079-1089.

Barrett, J., & Hamilton, W. (2008). Pathways to the diagnosis of lung cancer in the UK: a cohort study. *BMC Fam Pract, 9*, 31.

Barrett, J., Jiwa, M., Rose, P., & Hamilton, W. (2006). Pathways to the diagnosis of colorectal cancer: an observational study in three UK cities. *Fam Pract, 23*(1), 15−19.

Barrett, J., Sharp, D.J., Stapley, S., Stabb, C., Hamilton, W. (2010). Pathways to the diagnosis of ovarian cancer in the UK: a cohort study in primary care. *Bjog, 117*(5), 610−614.

Bosch, C., Vogel, S., Schrappe, M., Grischke, E.M., Seyfarth-Metzger, I., & Breitscheidel, L. (2010). Controlling the cost of care of breast cancer patients in germany using clinical pathways. *Value in Health, 13*(3), A49.

Brannstrom, M., Furst, C.J., Tishelman, C., Petzold, M., & Lindqvist, O. (2016). Effectiveness of the Liverpool care pathway for the dying in residential care homes: An exploratory, controlled before-and-after study. *Palliat Med, 30*(1), 54−63.

Chan, R.J., Webster, J., & Bowers, A. (2016). End-of-life care pathways for improving outcomes in caring for the dying. *Cochrane Database Syst Rev, 2*, CD008006.

Chang, P.L., Li, Y.C., & Lee, S.H. (2002). The differences in health outcomes between Web-based and paper-based implementation of a clinical pathway for radical nephrectomy. *BJU Int, 90*(6), 522−528.

Chang, P.L., Wang, T.M., Huang, S.T., Hsieh, M.L., Chuang, Y.C., & Chang, C.H. (2000). Improvement of health outcomes after continued implementation of a clinical pathway for radical nephrectomy. *World J Urol, 18*(6), 417−421.

Chang, S.S., Cookson, M.S., Baumgartner, R.G., Wells, N., & Smith, J.A., Jr. (2002). Analysis of early complications after radical cystectomy: results of a collaborative care pathway. *J Urol, 167*(5), 2012−2016.

Chang, S.S., Smith, J.A., Jr., Girasole, C., Baumgartner, R.G., Roth, B.J., & Cookson, M.S. (2002). Beneficial impact of a clinical care pathway in patients with testicular cancer undergoing retroperitoneal lymph node dissection. *J Urol, 168*(1), 87−92.

Chang, W.C., Lee, C.C., Wu, H.C., & Yeh, L.S. (2003). Laparoscopy-assisted vaginal hysterectomy clinical pathway. A multivariate analysis of impact on costs and quality of care. *Gynecol Obstet Invest, 55*(4), 231−234.

Chang, W.C., Li, T.C., & Lin, C.C. (2003). The effect of physician experience on costs and clinical outcomes of laparoscopic-assisted vaginal hysterectomy: a multivariate analysis. *J Am Assoc Gynecol Laparosc, 10*(3), 356−359.

Chase, D.M., Lopez, S., Nguyen, C., Pugmire, G.A., & Monk, B.J. (2008). A clinical pathway for postoperative management and early patient discharge: does it work in gynecologic oncology? *Am J Obstet Gynecol, 199*(5), 541.e1−7.

Choi, J.W., Xuan, Y., Hur, H., Byun, C.S., Han, S.U., & Cho, Y.K. (2013). Outcomes of Critical Pathway in Laparoscopic and Open Surgical Treatments for Gastric Cancer Patients: Patients Selection for Fast-Track Program through Retrospective Analysis. *J Gastric Cancer, 13*(2), 98−105.

Choi, J.Y. (2011). Treatment algorithm for intermediate and advanced stage hepatocellular carcinoma: Korea. *Oncology, 81* Suppl 1, 141−147.

Cohen, J., Stock, M., Andersen, P., & Everts, E. (1997). Critical pathways for head and neck surgery. Development and implementation. *Arch Otolaryngol Head Neck Surg, 123*(1), 11−14.

Costantini, M., Ottonelli, S., Canavacci, L., Pellegrini, F., Beccaro, M., & Group LCPRICTS. (2011). The effectiveness of the Liverpool care pathway in improving end of life care for dying cancer patients in hospital. A cluster randomised trial. *BMC Health Serv Res, 11*, 13.

Costantini, M., Pellegrini, F., Di Leo, S., Beccaro, M., Rossi, C., Flego, G., … Higginson, I.J. (2014). The Liverpool Care Pathway for cancer patients dying in hospital medical wards: a before-after cluster phase II trial of outcomes reported by family members. *Palliat Med, 28*(1), 10−17.

Costantini, M., Romoli, V., Leo, S.D., Beccaro, M., Bono, L., Pilastri, P., … Higginson, I.J. (2014). Liverpool Care Pathway for patients with cancer in hospital: a cluster randomised trial. *Lancet, 383*(9913), 226−237.

Dautremont, J.F., Rudmik, L.R., Nakoneshny, S.C., Chandarana, S.P., Matthews, T.W., Schrag, C., … Dort, M.D. (2016). Understanding the impact of a clinical care pathway for major head and neck cancer resection on postdischarge healthcare utilization. *Head Neck, 38* Suppl 1, E1216−1220.

Dautremont, J.F., Rudmik, L.R., Yeung, J., Asante, T., Nakoneshny, S.C., Hoy, M., … Dort, J.C. (2013). Cost-effectiveness analysis of a postoperative clinical care pathway in head and neck surgery with microvascular reconstruction. *J Otolaryngol Head Neck Surg, 42*, 59.

De Luc, K. (2000). Care pathways: an evaluation of their effectiveness*. Journal of Advanced Nursing, 32*(2), 12p.

Delgado, A., Kim, T., Kreys, E., Koeller, J.M., & Author, A., (2013). The University of Texas H, et al. The impact of compliance to oncology pathways that include G-CSF rules for use on ER visits/hospitalizations in a multistate program. *Journal of Clinical Oncology, 31*, 31 SUPPL. 1.

Dyrop, H.B., Safwat, A., Vedsted, P., Maretty-Nielsen, K., Hansen, B.H., Jorgensen, P.H., … Keller, J. (2013). Cancer Patient Pathways shortens waiting times and accelerates the diagnostic process of suspected sarcoma patients in Denmark. *Health Policy, 113*(1-2), 110−117.

Fackler-Schwalbe, I., & Spaeth-Schwalbe, E. (2014). Reimbursement of special medical oncology outpatient care. Cost accounting of transsectorally clinical pathways as a model for a new fee system. *Oncology Research and Treatment, 37*, 83.

Fields, K.K., Watson, A., Durand, A., Simpson, T., Stewart, S., Shi, J.Q., … Rollison, D.E. (2017). Using cancer registry data to improve adherence with breast cancer pathways. *Journal of Clinical Oncology, 35*, 8 Supplement 1.

Fiore, J.F., Jr., Bejjani, J., Conrad, K., Niculiseanu, P., Landry, T., Lee, L., … Feldman, L.S. (2016). Systematic review of the influence of enhanced recovery pathways in elective lung resection. *J Thorac Cardiovasc Surg, 151*(3), 708−715.e1.

Forrest, L.F., Sowden, S., Rubin, G., White, M., & Adams, J. (2017). Socio-economic inequalities in stage at diagnosis, and in time intervals on the lung cancer pathway from first symptom to treatment: systematic review and meta-analysis. *Thorax, 72*(5), 430−436.

Gasbarrini, A., Li, H., Cappuccio, M., Mirabile, L., Paderni, S., Terzi, S., & Boriani, S. (2010). Efficacy evaluation of a new treatment algorithm for spinal metastases. *Spine, 35*(15), 1466−1470.

Gerardi, M.A., Santillan, A., Meisner, B., Zahurak, M.L., Diaz Montes, T.P., Giuntoli, R.L. 2nd, & Bristow, R.E. (2008). A clinical pathway for patients undergoing primary cytoreductive surgery with rectosigmoid colectomy for advanced ovarian and primary peritoneal cancers. *Gynecol Oncol, 108*(2), 282−286.

Gordon, S.A., & Reiter, E.R. (2016). Effectiveness of critical care pathways for head and neck cancer surgery: A systematic review. *Head Neck, 38*(9), 1421−1427.

Grau, C., Christensen, A., Lyhne, N.M., Alanin, M.C., Bruun, M.T., Jung, T.H., … & Jespersen, J.B.B., on behalf of the Danish Society for Head and Neck Oncology (DSHHO) and The Danish Head and Neck Cancer Group (DAHANCA). (2011) Accelerated clinical pathways have caused a significant reduction in time for diagnosis and treatment of head and neck cancer in Denmark in 2010 compared to 2002. *European Journal of Cancer, 47*, S545.

Gustafsson, U.O., Hausel, J., Thorell, A., Ljungqvist, O., Soop, M., & Nygren, J. (2011). Adherence to the enhanced recovery after surgery protocol and outcomes after colorectal cancer surgery. *Arch Surg, 146*(5), 571−577.

Hingh, I.D.E., Demelinne, J., Rutten, H., Nienhuijs, S., Heesakkers, F., & Luyer, M. (2014). Faster, safer and more costeffective care for patients with pancreatic cancer after implementation of a preoperative pathway coordinated by nurse specialist. *Hpb, 16*, 376.

Hirasaki, S., Tanimizu, M., Moriwaki, T., Hyodo, I., Shinji, T., Koide, N., & Shiratori, Y. (2004). Efficacy of clinical pathway for the management of mucosal gastric carcinoma treated with endoscopic submucosal dissection using an insulated-tip diathermic knife. *Intern Med, 43*(12), 1120−1125.

Hoverman, J.R., Cartwright, T.H., Patt, D.A., Espirito, J.L., Clayton, M.P., Garey, J.S., … Beveridge, R.A. (2011). Pathways, outcomes, and costs in colon cancer: retrospective evaluations in two distinct databases. *J Oncol Pract, 7*(3 Suppl), 52s−59s.

Hsu, Y.C., Tsui, K.H., Chen, C.L., Lee, S.H., Wu, Y.S., & Chang, P.L. (2008). Web-based clinical pathway for reducing practice variations in radical prostatectomy. *Chang Gung Med J, 31*(6), 567−575.

Hubner, M., Addor, V., Slieker, J., Griesser, A.C., Lecureux, E., Blanc, C., & Demartines, N. (2015). The impact of an enhanced recovery pathway on nursing workload: A retrospective cohort study. *Int J Surg, 24*(Pt A), 45−50.

Husbands, J.M., Weber, R.S., Karpati, R.L., Weinstein, G.S., Chalian, A.A., Goldberg, A.N., … Wolf, P.F. (1999). Clinical care pathways: decreasing resource utilization in head and neck surgical patients. *Otolaryngol Head Neck Surg, 121*(6), 755−759.

Iijima, K., Nakagawa, K., Okumura, S., Sato, Y., & Tsuchiya, S. (2003). A clinical pathway for pulmonary resections with conventional thoracotomies in patients with neoplastic diseases. *Japanese Journal of Lung Cancer, 43*(2), 113−120.

Ise, Y., Honjo, K., So, S., Senoo, M., Katayama, S., Hirano, M., … Kiyama, T. (2003). Pharmacoeconomical evaluation of clinical pathway in gastrectomy patients. *Journal of the Nippon Medical School, 70*(1), 53−56.

Ishiguro, S., Yamamoto, S., Fujita, S., Akasu, T., Kobayashi, Y., & Moriya, Y. (2008). Effect of a clinical pathway after laparoscopic surgery for colorectal cancer. *Hepatogastroenterology, 55*(85), 1315−1319.

Jackman, D.M., Zhang, Y., Dalby, C., Nguyen, T., Nagle, J., Lydon, C.A., … Jacobson, J.O. (2017). Cost and Survival Analysis Before and After Implementation of Dana-Farber Clinical Pathways for Patients With Stage IV Non-Small-Cell Lung Cancer. *J Oncol Pract, 13*(4), e346−e352.

Jakobsen, J.K., & Jensen, J.B. (2016). DaPeCa-2: Implementation of fast-track clinical pathways for penile cancer shortens waiting time and accelerates the diagnostic process--A comparative before-and-after study in a tertiary referral centre in Denmark. *Scand J Urol, 50*(1), 80−87.

Jensen, K.H., & Maina, P.J. (2015). Cancer pathways are associated with improved long-term survival. *Dan Med J, 62*(2).

Kagan SH, Chalian AA, Goldberg AN, Rontal ML, Weinstein GS, Prior B, … Weber, R.S. (2002). Impact of age on clinical care pathway length of stay after complex head and neck resection. *Head Neck, 24*(6), 545−548; discussion 545.

Kardos, S.V., Chan, K.G., Yuh, B.E., Yamzon, J., Ruel, N., Zachariah, F., … Crocitto, L.E. (2016). Recovery after surgery and care coordination pathway at City of Hope: Length of stay, readmissions, and complications. *Journal of Clinical Oncology, 34*, 2 SUPPL. 1.

Katterhagen, G. (1996). Physician compliance with outcome-based guidelines and clinical pathways in oncology. Oncology (Williston), 10(11 Suppl), 113−121.

Keetch, D.W., & Buback, D. (1998). A clinical-care pathway for decreasing hospital stay after radical prostatectomy. *Br J Urol, 81*(3), 398−402.

Kennedy, E.P., Grenda, T.R., Sauter, P.K., Rosato, E.L., Chojnacki, K.A., Rosato, F.E., Jr., … Yeo, C.J. (2009). Implementation of a critical pathway for distal pancreatectomy at an academic institution. *Journal of gastrointestinal surgery : official journal of the Society for Surgery of the Alimentary Tract, 13*(5), 938−944.

Khan, S.A., Ullah, S., Ahmed, J., Wilson, T.R., McNaught, C., Hartley, J., & Macfie, J. (2013). Influence of enhanced recovery after surgery pathways and laparoscopic surgery on health-related quality of life. *Colorectal Dis, 15*(7), 900−907.

Khoo, C.K., Vickery, C.J., Forsyth, N., Vinall, N.S., & Eyre-Brook, I.A. (2007). A prospective randomized controlled trial of multimodal perioperative management protocol in patients undergoing elective colorectal resection for cancer. *Ann Surg, 245*(6), 867−872.

Kim, H.S., Kim, S.O., & Kim, B.S. (2015). Use of a clinical pathway in laparoscopic gastrectomy for gastric cancer. *World J Gastroenterol, 21*(48), 13507−13517.

Kiyama, T., Tajiri, T., Yoshiyuki, T., Mizutani, T., Okuda, T., Fujita, I., … Tokunaga, A. (2003). An economic evaluation on the clinical pathway for gastrectomy. [Japanese]. *Japanese Journal of Gastroenterology, 100*(5), 555−561.

Klinkhammer-Schalke, M., Koller, M., Steinger, B., Ehret, C., Ernst, B., Wyatt, J.C., … Lorenz, W. (2012). Direct improvement of quality of life using a tailored quality of life diagnosis and therapy pathway: randomised trial in 200 women with breast cancer. *Br J Cancer, 106*(5), 826−838.

Klinkhammer-Schalke, M., Lindberg, P., Koller, M., Wyatt, J.C., Hofstadter, F., Lorenz, W., & Steinger, B. (2015). Direct improvement of quality of life in colorectal cancer patients using a tailored pathway with quality of life diagnosis and therapy (DIQOL): study protocol for a randomised controlled trial. *Trials, 16*, 460.

Kobayashi, S. T., Diz, M.D.P.E., Campolina, A.G., De Soárez, P.C., Ribeiro, U. Jr, Nahas, S.C., … Hoff, P.M. (2017). Integrated care pathway for rectal cancer treatment: health care resource utilization, costs, and outcomes. *International Journal of Evidence-Based Healthcare, 15*(2), 10p.

Konety, B.R., Painter, L., & Bahnson, R.R.. (1996). A cost containment strategy for radical retropubic prostatectomy: Results from implementation of a clinical pathway program. *Urol, 2*(3), 80−87.

Kreys, E.D., & Koeller, J.M. (2013). Documenting the benefits and cost savings of a large multistate cancer pathway program from a payer's perspective. *J Oncol Pract, 9*(5), e241−247.

Langham, J., Langham, S., Weir, S., & Ralston, S. (2013). The use of European electronic health records to investigate cancer treatment pathways. *Value in Health, 16*(7), A615.

Lee, L., Li, C., Robert, N., Latimer, E., Carli, F., Mulder, D.S., … Feldman, L.S. (2013). Economic impact of an enhanced recovery pathway for oesophagectomy. *Br J Surg, 100*(10), 1326−1334.

Leibman, B.D., Dillioglugil, O., Abbas, F., Tanli, S., Kattan, M.W., & Scardino, P.T. (1998). Impact of a clinical pathway for radical retropubic prostatectomy. *Urology, 52*(1), 94−99.

Lemmens, L., Van Zelm, R., Borel Rinkes, I., Van Hillegersberg, R., & Kerkkamp, H. (2009). Clinical and organizational content of clinical pathways for digestive surgery: a systematic review. *Dig Surg, 26*(2), 91−99.

Letton, C., Cheung, C., & Nordin, A. (2013). Does an enhanced recovery integrated care pathway (ICP) encourage adherence to prescribing guidelines, accelerate postoperative recovery and reduce the length of stay for gynaecological oncology patients? *J Obstet Gynaecol, 33*(3), 296−297.,

Lindberg, P., Steinger, B., Koller, M., Furst, A., Kastel, M., Obermaier, R., … Klinkhammer‐Schalke, M. (2016). Implementing a clinical pathway with quality of life diagnosis and therapy of patients with primary colorectal cancer: A randomised controlled trial. *Oncology research and treatment, 39*, 137.

Llobera, J., Sansó, N., Ruiz, A., Llagostera, M., Serratusell, E., Serrano, C., … Leiva, A. (2017). Strengthening primary health care teams with palliative care leaders: protocol for a cluster randomized clinical trial. *BMC Palliative Care, 17*, 8p.

Lorenz, W., Klinkhammer-Schalke, M., Ernst, B., Jakob, C., Steinger, B., Hofstadter, F., & Koller, M. (2005). Implementing a clinical pathway for quality-of-life diagnostics and therapy in follow-up of breast cancer patients. *J Clin Oncol, 23*(16_suppl), 758.

Lyhne, N.M., Christensen, A., Alanin, M.C., Bruun, M.T., Jung, T.H., Bruhn, M.A., … Grau, C. (2013). Waiting times for diagnosis and treatment of head and neck cancer in Denmark in 2010 compared to 1992 and 2002. *European Journal of Cancer, 49*(7), 1627−1633.

Markar, S.R., Karthikesalingam, A., & Low, D.E. (2015). Enhanced recovery pathways lead to an improvement in postoperative outcomes following esophagectomy: systematic review and pooled analysis. *Dis Esophagus, 28*(5), 468−475.

Markar, S.R., Schmidt, H., Kunz, S., Bodnar, A., Hubka, M., & Low, D.E. (2014). Evolution of standardized clinical pathways: refining multidisciplinary care and process to improve outcomes of the surgical treatment of esophageal cancer. *J Gastrointest Surg, 18*(7), 1238−1246.

Maruyama, R., Miyake, T., Kojo, M., Aoki, Y., Suemitsu, R., Okamoto, T., … Ichinose, Y. (2006). Establishment of a clinical pathway as an effective tool to reduce hospitalization and charges after video-assisted thoracoscopic pulmonary resection. *Jpn J Thorac Cardiovasc Surg, 54*(9), 387−390.

Mashhad University of Medical S. (2014). A clinical trial on the effect of critical pathway method in Colorectal cancer Patients [Trial Registry Record]. Irct2013073014225N

McCray, D.K., Grobmyer, S.R., & Pederson, H.J. (2017). Impact of value based breast cancer care pathway implementation on pre-operative breast magnetic resonance imaging utilization. *Gland surg, 6*(1), 57−63.

Mori, T., Nishi, W., Yamada, T., Motooka, Y., Ikeda, K., Shiraiishi, K., & Suzuki, M. (2017). Regional clinical pathway for lung cancer in Kumamoto University Hospital. *Journal of Thoracic Oncology, 12* (1 Supplement 1), S1113-S1114.

Muehling, BM., Halter, GL., Schelzig, H., Meierhenrich, R., Steffen, P., Sunder-Plassmann, L., & Orend, KH. (2008). Reduction of postoperative pulmonary complications after lung surgery using a fast track clinical pathway. *European Journal of Cardio-thoracic Surgery, 34*, 174-180.

Munitiz, V., Martinez-de-Haro, L.F., Ortiz, A., Ruiz-de-Angulo, D., Pastor, P., & Parrilla, P. (2010). Effectiveness of a written clinical pathway for enhanced recovery after transthoracic (Ivor Lewis) oesophagectomy. *Br J Surg, 97*(5), 714−718.

Neo, P.S., Poon, M.C., Peh, T.Y., Ong, S.Y., Koo, W.H., Santoso, U., … Yee, A.C. (2012). Improvements in end-of-life care with a protocol-based pathway for cancer patients dying in a Singapore hospital. *Ann Acad Med Singapore, 41*(11), 483−493.

Nunez Mora, C., Chamorro Ramos, L., Rendon Sanchez, D., Rios Gonzalez, E., Pastor Arquero, T., Aguilera Bazan, A., … de la Peña Barthel, J.J. (2001). Clinical pathway for TUR of bladder neoplasms. Analysis of the first 2 years of its implementation. [Spanish]. *Archivos espanoles de urologia, 54*(8), 777−786.

Nussbaum, D.P., Penne, K., Speicher, P.J., Stinnett, S.S., Perez, A., White, R.R., … Blazer, D.G. 3rd. (2014). The role of clinical care pathways: an experience with distal pancreatectomy. *J Surg Res, 190*(1), 64−71.

O'Connell, D.A., Barber, B., Klein, M.F., Soparlo, J., Al-Marzouki, H., Harris, J.R., & Seikaly, H. (2015). Algorithm based patient care protocol to optimize patient care and inpatient stay in head and neck free flap patients. *J Otolaryngol Head Neck Surg, 44*, 45.

Ogawa, T., Terada, A., Yamada, Y., Ijichi, K., Hasegawa, Y., & Fujimoto, Y. (2004). The meaning clinical pathway of the operation for thyroid tumor and parotid tumor. [Japanese]. *Practica Oto-Rhino-Laryngologica, 97*(6), 555−561.

Oh, J.K., Park, N.H., & Oh, S.J. (2014). Effect of the systematised critical pathway protocol on emptying failure as a secondary complication of radical hysterectomy due to uterine cervix cancer. *J Clin Nurs, 23*(11-12), 1702−1707.

Ortega-Lucea, S.M., Martinez-Ubieto, J., Judez-Legaristi, D., Munoz-Rodriguez, L., Gil-Bona, J., & Pascual-Bellosta, A.M. (2015). The results of implementing a fast-track protocol in radical cystectomy in a tertiary hospital. *Actas Urol Esp, 39*(10), 620−627.

Page, B.J., Hanks, A., Marshall, H., Yang, I.A., Bowman, R., & Fong, K. (2013). A comparison of the lung cancer journey-metropolitan and non-metropolitan. *Journal of Thoracic Oncology, 8*, S706.

Pease, N.J., Harris, R.J., & Finlay, I.G. (2004). Development and audit of a care pathway for the management of patients with suspected malignant spinal cord compression. *Physiotherapy, 90*(1), 27−34.

Pellino, G., Simillis, C., Kontovounisios, C., Baird, D.L., Nikolaou, S., Warren, O., … Rasheed, S. (2017). Colorectal cancer diagnosed during pregnancy: systematic review and treatment pathways. *Eur J Gastroenterol Hepatol, 29*(7), 743−753.

Pengfei, L., Weidong, J., Geliang, X., Jinliang, M. Guangyao, L., Ya, C., Jihai, Y. & Wenbin, L. (2015). Clinical pathway of enhanced recovery after surgery in patients undergoing hepatectomy for primary liver cancer. *Chinese Journal of General Surgery, 30*(11), 862−865.

Peters, M., & Eckert, M. (2016). Implementing optimal cancer care pathways; lessons learnt from the evidence to improve cancer care outcomes: A systematic review. *Supportive Care in Cancer, 24*(1 Supplement 1), S245.

Pfau, P.R., Cooper, G.S., Carlson, M.D., Chak, A., Sivak, M.V., Gonet, J.A., … Wong, R.C. (2004). Success and shortcomings of a clinical care pathway in the management of acute nonvariceal upper gastrointestinal bleeding. *American Journal of Gastroenterology, 99*(3), 425−431.

Pirnejad, H., Gao, C., Reddingius, R., Rijneveld, A., & Bal, R. (2013). Improving chemotherapy processes with a protocol-based information system: a pre and post-implementation study. *Int J Med Inf, 82*(4), 220−229.

Porteous, G.H., Neal, J.M., Slee, A., Schmidt, H., & Low, D.E. (2015). A standardized anesthetic and surgical clinical pathway for esophageal resection: impact on length of stay and major outcomes. *Reg Anesth Pain Med, 40*(2), 139−149.

Porter, G.A., Pisters, P.W., Mansyur, C., Bisanz, A., Reyna, K., Stanford, P., … Evans, D.B. (2000). Cost and utilization impact of a clinical pathway for patients undergoing pancreaticoduodenectomy. *Ann Surg Oncol, 7*(7), 484−489.

Preston, S.R., Markar, S.R., Baker, C.R., Soon, Y., Singh, S., & Low, D.E. (2013). Impact of a multidisciplinary standardized clinical pathway on perioperative outcomes in patients with oesophageal cancer. *Br J Surg, 100*(1), 105−112.

Quraishi, T., Panchagnula, U., & Bansal, S. (2012). Economic impact of multi-disciplinary pathway for uro-oncology surgical services in a tertiary teaching hospital in the United Kingdom. *Anesthesia and Analgesia, 1*, S183.

Raman, V., Kaiser, L.R., & Erkmen, C.P. (2016). Clinical pathway for esophagectomy improves perioperative nutrition. *Healthc (Amst), 4*(3), 166−172.

Rashid, O.M., Pimiento, J.M., Gamenthaler, A.W., Nguyen, P., Ha, T.T., Hutchinson, T., … Malafa, M.P. (2016). Outcomes of a Clinical Pathway for Borderline Resectable Pancreatic Cancer. *Ann Surg Oncol, 23*(4), 1371−1379.

Rich-Ruiz, M., Requena-Tapia. M., Regueiro-López, J.C., López-Luque, A.J., Muñoz-Gomáriz, E., & Prieto-Rodríguez, M. (2006). Impact of a clinical pathway in cases of transurethral resection of the prostate and bladder. *Enfermeria Clinica, 16*(5), 11p.

Richter-Ehrenstein, C., Heymann, S., Schneider, A., & Vargas Hein, O. (2012). Effects of a clinical pathway 3 years after implementation in breast surgery. *Arch Gynecol Obstet, 285*(2), 515−520.

Roberts, H.C., Pickering, R.M., Onslow, E., Clancy, M., Powell, J., Roberts, A., … Bray, J. (2004). The effectiveness of implementing a care pathway for femoral neck fracture in older people: a prospective controlled before and after study. *Age Ageing, 33*(2), 178−184.

Sancho, C., Villalba, F.L., Garcia-Coret, M.J., Vazquez, A., Safont, M.J., Hernandez, A., … Roig, J.V. (2010). Self-evaluation of a clinical pathway to improve the results of rectal cancer. [Spanish]. *Cirugia Espanola, 87*(4), 231−238.

Santillan, A., Govan, L., Zahurak, M.L., Diaz-Montes, T.P., Giuntoli, R.L., 2nd, & Bristow, R.E. (2008). Feasibility and economic impact of a clinical pathway for pap test utilization in Gynecologic Oncology practice. *Gynecol Oncol, 109*(3), 388−393.

Santoso, U., Iau, P.T.C., Lim, J., Koh, C.S., & Pang, Y.T. (2002). The mastectomy clinical pathway: what has it achieved? *Ann Acad Med Singapore, 31*(4), 440-445.

Schwarzbach, M., Rossner, E., Schattenberg, T., Post, S., Hohenberger, P., & Ronellenfitsch, U. (2010). Effects of a clinical pathway of pulmonary lobectomy and bilobectomy on quality and cost of care. *Langenbecks Arch Surg, 395*(8), 1139−1146.

Schwarzbach, M.H., Ronellenfitsch, U., Wang, Q., Rossner, E.D., Denz, C., Post, S., & Hohenberger, P. (2010). Effects of a clinical pathway for video-assisted thoracoscopic surgery (VATS) on quality and cost of care. *Langenbeck's archives of surgery, 395*(4), 333−340.

Scott, J.A., Milligan, S., Wong, W., Winn, D., Cooper, J., Schneider, N., … Feinberg, B.A. (2013). Validation of observed savings from an oncology clinical pathways program. *Journal of Clinical Oncology Conference, 31*, 15 SUPPL. 1.

Seo, H.S., Song, K.Y., Jeon, H.M., & Park, C.H. (2012). The impact of an increased application of critical pathway for gastrectomy on the length of stay and cost. *J Gastric Cancer, 12*(2), 126−131.

Shamji, F.M., & Deslauriers, J. (2013). Fast-tracking investigation and staging of patients with lung cancer. *Thorac Surg Clin, 23*(2), 187−191.

Sherman, D., Matthews, T.W., Lampe, H., & LeBlanc, S. (2001). Laryngectomy clinical pathway: development and review. *J Otolaryngol, 30*(2), 115−120.

Shetiwy, M., Fady, T., Shahatto, F., & Setit, A. (2017). Standardizing the Protocols for Enhanced Recovery From Colorectal Cancer Surgery: Are We a Step Closer to Ideal Recovery? *Ann Coloproctol, 33*(3), 86−92.

Sivakumaran, Y., Ng, K.S., Bhan, C., Nassar, N., & Gladman, M.A. (2013). An evaluation of the colorectal cancer referral pathway and adherence to Australian guidelines in a tertiary referral centre. *Journal of Gastroenterology and Hepatology, 28*, 144.

So, J.B., Lim, Z.L., Lin, H.A., & Ti, T.K. (2008). Reduction of hospital stay and cost after the implementation of a clinical pathway for radical gastrectomy for gastric cancer. *Gastric Cancer, 11*(2), 81−85.

Song, X.P., Tian, J.H., Cui, Q., Zhang, T.T., Yang, K.H., & Ding, G.W. (2014). Could clinical pathways improve the quality of care in patients with gastrointestinal cancer? A meta-analysis. *Asian Pac J Cancer Prev, 15*(19), 8361−8366.

Soria-Aledo, V., Mengual-Ballester, M., Pellicer-Franco, E., Carrillo-Alcaraz, A., Cases-Baldo, M.J., Carrasco-Prats, M., … Aguayo-Albasini, J.L. (2011). Evaluation of a clinical pathway to improve colorectal cancer outcomes. *Am J Med Qual, 26*(5), 396−404.

Stephen, A.E., & Berger, D.L. (2003). Shortened length of stay and hospital cost reduction with implementation of an accelerated clinical care pathway after elective colon resection. *Surgery, 133*(3), 277−282.

Stocker, R., & Close, H. (2013). Assessing the uptake of the Liverpool Care Pathway for dying patients: a systematic review. *BMJ support, 3*(4), 399−404.

Takagi, J. (1996). Developing and implementing a critical care pathway for radical prostatectomy. [presentation]. *Journal of Oncology Pharmacy Practice, 2*(1), 61-64.

Tateno, Y., & Ishikawa, S. (2012). Clinical pathways can improve the quality of pain management in home palliative care in remote locations: retrospective study on Kozu Island, Japan. *Rural Remote Health, 12*, 1992.

Tomaszek, S.C., Cassivi, S.D., Allen, M.S., Shen, K.R., Nichols, F.C. 3rd, Deschamps, C., & Wigle, D.A. (2010). An alternative postoperative pathway reduces length of hospitalisation following oesophagectomy. *Eur J Cardiothorac Surg, 37*(4), 807−813.

Tomiki, Y., Kawai, M., Takehara, K., Tashiro, Y., Munakata, S., Kure, K., … Sakamoto, K. (2015). Clinical pathway to discharge 3 days after colorectal endoscopic submucosal dissection. *Dig Endosc, 27*(6), 679−686.

Turini, I.G., Clark, M., Tucci, C., Machan, J., Golijanin, D., Pareek, G., & Renzulli, J. (2016). Reduced length of stay and readmission rates using a standardized clinical care pathway following robotic prostatectomy*. Journal of Urology, 195*, 4S, e86.

Una, E., & Lopez-Lara, F. (2010). Pilot study of a clinical pathway implementation in rectal cancer. *Clin Med Insights Oncol, 4*, 111−115.

Valentin-Lopez, B., Ferrandiz-Santos, J., Blasco-Amaro, J.A., Morillas-Sainz, J.D., & Ruiz-Lopez, P. (2012). Assessment of a rapid referral pathway for suspected colorectal cancer in Madrid. *Family practice, 29*(2), 182−188.

Vallverdú-Cartié, H., Comajuncosas-Camp, J., Orbeal-Sáenz, R.A., López-Negre, J.L., Gris Garriga, P.J., Jimeno-Fraile, J., … Parés, D. (2011). Results of implementation of a fast track pathway for diagnosis of colorectal cancer. *Rev Esp Enferm Dig, 103*(8), 402−407.

Van Beek, K., Siouta, N., Preston, N., Hasselaar, J., Hughes, S., Payne, S., … Menten, J. (2016). To what degree is palliative care integrated in guidelines and pathways for adult cancer patients in Europe: a systematic literature review. *BMC Palliat Care, 15*, 26.

Van Dam, P.A., Verheyden, G., Sugihara, A., Trinh, X.B., Van Der Mussele, H., Wuyts, H., … Dirix, L. (2013). A dynamic clinical pathway for the treatment of patients with early breast cancer is a tool for better cancer care: implementation and prospective analysis between 2002-2010. *World J Surg Oncol, 11*, 70.

Van Hoeve, J., de Munck, L., Otter, R., de Vries, J., & Siesling, S. (2014). Quality improvement by implementing an integrated oncological care pathway for breast cancer patients. *Breast, 23*(4), 364−370.

Van Hoeve, J.C., Elferink, M.A., Klaase, J.M., Kouwenhoven, E.A., Schiphorst, P.P., & Siesling, S. (2015). Long-term effects of a regional care pathway for patients with rectal cancer. *Int J Colorectal Dis, 30*(6), 787−795.

Van Houdt, S., Heyrman, J., Vanhaecht, K., Sermeus, W., & De Lepeleire, J. (2013). Care pathways to improve care co-ordination and quality between primary and hospital care for patients with radical prostatectomy: a quality improvement project. *Qual Prim Care, 21*(3), 149−155.

Van Zelm, R., Janssen, I., Vanhaecht, K., de Buck van Overstraeten, A., Panella, M., Sermeus, W., & Coeckelberghs, E. (2018). Development of a model care pathway for adults undergoing colorectal cancer surgery: Evidence-based key interventions and indicators. *J Eval Clin Pract, 24*(1), 232-239.

Varela-Centelles, P., Seoane, J., Lopez-Cedrun, J.L., Fernandez-Sanroman, J., Garcia-Martin, J.M., Takkouche, B., … Seoane-Romero, J.M. (2018). The length of patient and primary care time interval in the pathways to treatment in symptomatic oral cancer. A quantitative systematic review. *Clin Otolaryngol, 43*(1), 164-171.

Verhofstede, R., Smets, T., Cohen, J., Noortgate, N., Heide, A., & Deliens, L. (2012). Study protocol for a cluster randomized trial to evaluate the influence of the liverpool care pathway on end-of-life care in acute geriatric hospital wards in flanders. *Palliative medicine, 26*(4), 548−549.

Wang, M.L., Chen, W.Q., Li, Y.J., Wang, Y.X., & Li, Y.Q. (2017). Implementation and improvement of clinical pathway for colorectal polyps. [Chinese]. *World Chinese Journal of Digestology, 25*(6), 546−551.

Wu, C.L., Benson, A.R., Hobson, D.B., Roda, C.P., Demski, R., Galante, D.J., … Wick, E.C. (2015). Initiating an Enhanced Recovery Pathway Program: An Anesthesiology Department's Perspective. *Jt Comm J Qual Patient Saf, 41*(10), 447−456.

Xuping, S., Jinhui, T., Qi, C., Guowu, D., Kehu, Y., & Peizhen, Z. (2014). Effects of clinical pathways used in surgery for uterine fibroids: a meta-analysis. *J Cancer Res Ther, 10*(1), 180−186.

Yetzer, J.G., Pirgousis, P., Li, Z., & Fernandes, R. (2017). Clinical Pathway Implementation Improves Efficiency of Care in a Maxillofacial Head and Neck Surgery Unit. *J Oral Maxillofac Surg, 75*(1), 190-196.

Yeung, J.K., Dautremont, J.F., Harrop, A.R., Asante, T., Hirani, N., Nakoneshny, S.C., … Dort, J.C. (2014). Reduction of pulmonary complications and hospital length of stay with a clinical care pathway after head and neck reconstruction. *Plast Reconstr Surg, 133*(6), 1477−1484.

Yueh, B., Weaver, E.M., Bradley, E.H., Krumholz, H.M., Heagerty, P., Conley, A., & Sasaki, C.T. (2003). A critical evaluation of critical pathways in head and neck cancer. *Arch Otolaryngol Head Neck Surg, 129*(1), 89−95.

Zauner, G., Popper, N., & Breitenecker, F. (2014). Assessment of Lung Cancer Treatment by Disease Phase Using National Cancer Registry Data Linked with Treatment Pathway cost Data in Austria. *Value Health, 17*(7), A653.

Zhang, Y., Fraile, B., Dalby, C.K., Nguyen, T., Nagle, J., Lydon, C.A., … Jackman, D.M. (2017). Cost and survival analysis before and after implementation of Dana-Farber Clinical Pathways for Patients with Stage IV Non-Small Cell Lung Cancer. *Journal of Clinical Oncology, 35*(8_suppl), 3−3.

Zhu, L., Li, J., Li, X.K., Feng, J.Q., & Gao, J.M. (2014). Impact of a clinical pathway on hospital costs, length of stay and early outcomes after hepatectomy for hepatocellular carcinoma. *Asian Pac J Cancer Prev, 15*(13), 5389−5393.
