# Supplementary material for: Effects of oncological care pathways in primary and secondary care on patient, professional and health systems outcomes: a systematic review and meta-analysis
Source: Syst Rev. 2020 Oct 25;9:246. doi: 10.1186/s13643-020-01498-0 (PMC7586678; doi:10.1186/s13643-020-01498-0)
Supplement: Supplementary file 6 — Additional file 6. Risk of bias of included studies. [file 13643_2020_1498_MOESM6_ESM.docx]

**Additional file 6 Risk of Bias of included studies**

Table Risk of bias for randomized controlled trials-RCT’s-, and non-randomized controlled trials-NRCTs-, see below

|  |  | **Risk of bias** | | | | | | |  | |  |  | **Overall**  **score*** | |  |
| --- | --- | --- | --- | --- | --- | --- | --- | --- | --- | --- | --- | --- | --- | --- | --- |
|  | Study ID | Was the allocation sequence adequately generated? | Was the allocation adequately concealed? | Were baseline outcome measurements similar? | Were baseline characteristics similar? | Were incomplete outcome data adequately addressed? | Was knowledge of the allocated interventions adequately prevented during the study? | Was the study adequately protected against contamination? | | Was the study free from selective outcome reporting? | | Was the study free from other risk of bias? | | **High or**  **Unclear or**  **Low Risk** | |
| 1 | Chen et al. 2000 [21] | + | + | ? | ? | - | + | ? | | - | | - | | **High risk** | |
| 2 | Dahl et al. 2017 [29] | + | + | - | + | ? | + | - | | - | | - | | **High risk** | |
| 3 | Gendron et al. 2002 [22] | + | + | ? | + | - | - | - | | - | | - | | **High risk** | |
| 4 | Ghosh et al. 2001 [23] | + | + | - | + | ? | ? | - | | - | | - | | **High risk** | |
| 5 | Jeong et al. 2011 [24] | + | + | ? | - | - | - | ? | | - | | - | | **High risk** | |
| 6 | Kiyama et al, 2003 [25] | + | + | ? | + | + | ? | ? | | + | | + | | **Low risk** | |
| 7 | Tastan et al. 2012 [26] | + | + | ? | - | - | - | ? | | - | | - | | **High risk** | |
| 8 | Williams et al. 2015 [27] | - | ? | - | - | - | - | - | | - | | - | | **Low risk** | |

“-“ is low risk, “?” is unclear risk, “+” is high risk of bias.

* We will consider studies with low risk of bias for all key domains or where it seems unlikely for bias to seriously alter the results. We will consider studies where risk of bias in at least one domain is unclear or judged to have some bias that could raise doubts about the conclusions as having an unclear risk of bias. We will consider studies with a high risk of bias in at least one domain or judged to have serious bias that decreases the certainty of the conclusions as having a high risk of bias (Higgins, 2011).
