# Supplementary material for: Effects of oncological care pathways in primary and secondary care on patient, professional and health systems outcomes: a systematic review and meta-analysis
Source: Syst Rev. 2020 Oct 25;9:246. doi: 10.1186/s13643-020-01498-0 (PMC7586678; doi:10.1186/s13643-020-01498-0)
Supplement: Supplementary file 7 — Additional file 7. Quality assessment of cost evaluation studies. [file 13643_2020_1498_MOESM7_ESM.docx]

**Additional file 7 Quality assessment of cost evaluation studies, based on the Evers checklist available in the Cochrane Handbook**

|  |  | **Chen et al.**  **2000 [21]** | **Gendron et al.**  **2002 [22]** | **Ghosh et al.**  **2001 [23]** | **Jeong et al.**  **2011 [24]** | **Kiyama et al.**  **2003 [25]** | **Williams et al.**  **2015 [27]** |
| --- | --- | --- | --- | --- | --- | --- | --- |
| 1 | Is the study population clearly described? | y | y | n | y | y | y |
| 2 | Are competing alternatives clearly described? | n | n | n | y | y | y |
| 3 | Is a well-defined research question posed in answerable form? | n | n | n | n | y | n |
| 4 | Is the economic study design appropriate to the stated objective? | y | y | y | y | y | y |
| 5 | Is the chosen time horizon appropriate to include relevant costs and consequences? | y | y | y | y | y | y |
| 6 | Is the actual perspective chosen appropriate? | y | y | y | y | y | y |
| 7 | Are all important and relevant costs for each alternative identified? | y | y | y | y | y | y |
| 8 | Are all costs measured appropriately in physical units? | y | y | y | n | y | n |
| 9 | Are costs valued appropriately? | y | y | y | y | y | y |
| 10 | Are all important and relevant outcomes for each alternative identified? | y | y | y | y | y | y |
| 11 | Are all outcomes measured appropriately? | y | y | y | y | y | y |
| 12 | Are outcomes valued appropriately? | y | y | y | y | y | y |
| 13 | Is an incremental analysis of costs and outcomes of alternatives performed? | n | n | n | n | n | n |
| 14 | Are all future costs and outcomes discounted appropriately? | n | n | n | n | n | n |
| 15 | Are all important variables, whose values are uncertain, appropriately subjected to sensitivity analysis? | n | n | n | n | n | n |
| 16 | Do the conclusions follow from the data reported? | y | y | y | y | y | y |
| 17 | Does the study discuss the generalizability of the results to other settings and patient/ client groups? | n | n | n | y | n | n |
| 18 | Does the article indicate that there is no potential conflict of interest of study researcher(s) and funder(s)? | y | y | y | y | y | y |
| 19 | Are ethical and distributional issues discussed appropriately? | n | n | n | n | n | n |
