# Supplementary material for: Effects of oncological care pathways in primary and secondary care on patient, professional and health systems outcomes: a systematic review and meta-analysis
Source: Syst Rev. 2020 Oct 25;9:246. doi: 10.1186/s13643-020-01498-0 (PMC7586678; doi:10.1186/s13643-020-01498-0)
Supplement: Supplementary file 8 — Additional file 8. Original reported costs / charges data. [file 13643_2020_1498_MOESM8_ESM.docx]

**Additional file 8 Original reported costs / charges data**

| **Study ID** | **Country** | **Currency** | **Costs included** | **Pathway** | **Control** | **Reduction of costs,**  **per patient** | **P value** |
| --- | --- | --- | --- | --- | --- | --- | --- |
| **Chen et al. 2000 [21]** | USA | US$ | Total costs including hospital and professional fees:  surgery-related costs, treatment-related costs, medications, consultations, and assessment and diagnostic tests. | $6,227 | $8,459 (historical control group, HCG)  $6,885 (non-pathway group, NPG) | HCG vs pathway: -$2,232  NPG vs pathway: -$658 | p<0.001* |
| **Gendron et al. 2002 [22]** | USA | US$ | The charge summary was divided into the following 6 categories: total, hospital room, pharmacy, operating room, laboratory, and other charges. Professional fees were not included. | $78930 (>1 year, group 1)  $65919 (>3 years, group 2) | $105410 | Control vs pathway group 1: -$26480  Control vs pathway group 2: -$39491 |  |
| **Ghosh et al. 2001 [23]** (cervical cancer) | USA | US$ | Direct costs were obtained including hospitalization, pharmacy, laboratory, operation room, radiological, and other miscellaneous costs (the last includes: physical therapy, respiratory therapy, patient monitoring, and patient education). | $3,920 | $5,545 | -$1,625 (-29%) | - |
| **Ghosh et al. 2001 [23]** (endometrial cancer) | USA | US$ |  | $3,790 | $4,766 | -$976 (-32%) | - |
| **Jeong et al. 2011 [24]** (advanced gastric cancer) | Korea | KRW | Total hospital costs  There is no description available of which costs are included. | 7,359,437 Won | 7,384,470 Won | -25,033 Won | p=0.96 |
|  |  |  | Preoperative costs | 1,053,341 Won | 1,307,558 Won | -254,217 Won | p=0.00* |
|  |  |  | Postoperative costs | 6,306,096 Won | 6,079,912 Won | +226,184 Won | p=0.64 |
| **Jeong et al. 2011 [24]** (early gastric cancer) | Korea | KRW | Total hospital costs  There is no description available of which costs are included. | 7,913,477 Won | 8,801,131 Won | -887,654 Won | p=0.08* |
|  |  |  | Preoperative costs | 1,167,514 Won | 1,564,053 Won | -396,539 Won | p=0.00* |
|  |  |  | Postoperative costs | 6,745,964 Won | 7,237,078 Won | -491,114 Won | P=0.27 |
| **Kiyama et al. 2003 [25]** | Japan | JPY | The total costs  The total direct costs reported were  the total medical costs (including medication and examinations). | 1502587 Yen | 1932197 Yen | -429610 yen | p<0.001 |
|  |  |  | Medication costs only | 190339 Yen | 270631 Yen | -80292 yen | p<.001 |
|  |  |  | Daily total costs | 58383 Yen | 55651 Yen | +2732 yen | N.S. |
| **Williams et al. 2015 [27]** | UK | GBP | Costs included: analgesic drug costs, pain clinic visits, use of physiotherapy, psychology and other resources. | £ 430 (640) | £ 230 (450) | +£200 | - |

Abrreviations: USA: United States of America; US$: United States Dollar; KRW: Korean Won; JPY: Japanese yen; N.S.: not significant; UK: United Kingdom; GBP: Pound sterling.
